# Deconstructing a multiple antibiotic resistance regulation through the quantification of its input function

**DOI:** 10.1038/s41540-017-0031-2

**Published:** 2017-10-06

**Authors:** Guillermo Rodrigo, Djordje Bajić, Ignacio Elola, Juan F. Poyatos

**Affiliations:** 1 Instituto de Biología Molecular y Celular de Plantas, CSIC–UPV, 46022 Valencia, Spain; 2 Logic of Genomic Systems Laboratory, CNB–CSIC, 28049 Madrid, Spain; 3 Present Address: Department of Ecology and Evolutionary Biology, Yale University, New Haven, USA

## Abstract

Many essential bacterial responses present complex transcriptional regulation of gene expression. To what extent can the study of these responses substantiate the logic of their regulation? Here, we show how the input function of the genes constituting the response, i.e., the information of how their transcription rates change as function of the signals acting on the regulators, can serve as a quantitative tool to deconstruct the corresponding regulatory logic. To demonstrate this approach, we consider the multiple antibiotic resistance (*mar*) response in *Escherichia coli*. By characterizing the input function of its representative genes in wild-type and mutant bacteria, we recognize a dual autoregulation motif as main determinant of the response, which is further adjusted by the interplay with other regulators. We show that basic attributes, like its reaction to a wide range of stress or its moderate expression change, are associated with a strong negative autoregulation, while others, like the buffering of metabolic signals or the lack of memory to previous stress, are related to a weak positive autoregulation. With a mathematical model of the input functions, we identify some constraints fixing the molecular attributes of the regulators, and also notice the relevance of the bicystronic architecture harboring the dual autoregulation that is unique in *E. coli*. The input function emerges then as a tool to disentangle the rationale behind most of the attributes defining the *mar* phenotype. Overall, the present study supports the value of characterizing input functions to deconstruct the complexity of regulatory architectures in prokaryotic and eukaryotic systems.

## Dissecting cellular “recipes”: Reverse engineering the complex regulation of a bacterial response with the use of input functions

Many cellular responses result from the integration of numerous regulatory signals. To deconstruct the regulation of one of these responses, which enables resistance to multiple antibiotics in *Escherichia coli*, a team led by Juan F. Poyatos at the National Center for Biotechnology in Madrid studied the response input function by combining theoretical models and experiments. This function quantifies the rate of transcription of the genes constituting the response with respect to the signals acting on its cognate regulators. By examining how the shape of the function changes in different situations, e.g., when a given regulator is mutated, the team identified the implications for the specificity and dynamics of the response of a dual autoregulation at the core of the control architecture. The use of input functions as quantitative tools allows us to reverse engineer the complex regulations that dictate essential physiological functions in both prokaryotic and eukaryotic cells.

## Introduction

Studies on the transcriptional control of gene expression date back to the early work by Jacob, Lwoff, Monod, and coworkers, who described how bacteria and viruses are able to adjust their metabolism or modify their lifestyle by regulating expression.^1,2^ Later efforts identified explicit DNA sequences involved in the regulation, with the characterization of the binding sites for both RNA polymerase, and regulatory proteins.^3^ Additional findings revealed cooperative and noncooperative interactions among regulators,^4^ and the possibility that the very constituents of an expression program could influence its control, i.e., autogenous regulation.^5^ All these features eventually resolved the function of *cis*-acting sequences as transducers of environmental information, from the activity of regulatory and RNA polymerase proteins to the precise expression pattern of the corresponding gene (see ref. 6 for a review of the implications of expression regulation in development and evolution).

After the accumulation of molecular details on these elements over the years, the study of regulation has focused on understanding the complex combinatorial action of many regulatory proteins.^6,7^ This issue generally involves an approach that merges the development of accurate mathematical modelling^8^ with new quantitative techniques for monitoring the dynamics of expression. Of particular note is the use of fluorescent proteins, which make possible to observe the activity of bacterial promoters in a non-destructive fashion (in contrast, for example, to the classical use of substrates for the *lacZ* gene).^9,10^

Some features of the new methodology are inspired by those applied in the computational or physical sciences: for example, when the study of a certain device is carried out by characterizing its output in response to a set of input signals. In the context of *cis-*regulatory sequences, the goal is to establish the strength of the transcription rate of a particular gene with respect to signals that alter the concentration of its active regulators: the so-called *input function* (also termed gene-regulation or dose-response function).^8,11^ Input functions do not necessarily follow the computational null models (i.e., AND, OR gates when considering two incoming signals^12^) but could present a new set of signal processing rules,^11^ which appear to be very plastic.^13^ Complementary work, in this context, examined how detailed expression patterns arise from a mixture of local (specific) and general (master) regulatory elements,^14^ and how the type of regulation, i.e., activation or repression, directs to more robust computations that better tolerate regulatory errors.^15^ Other issues like the control of cross talk among signals,^16^ or the presence of memory in gene expression^17^ have been recently addressed.

**Fig. 1:**
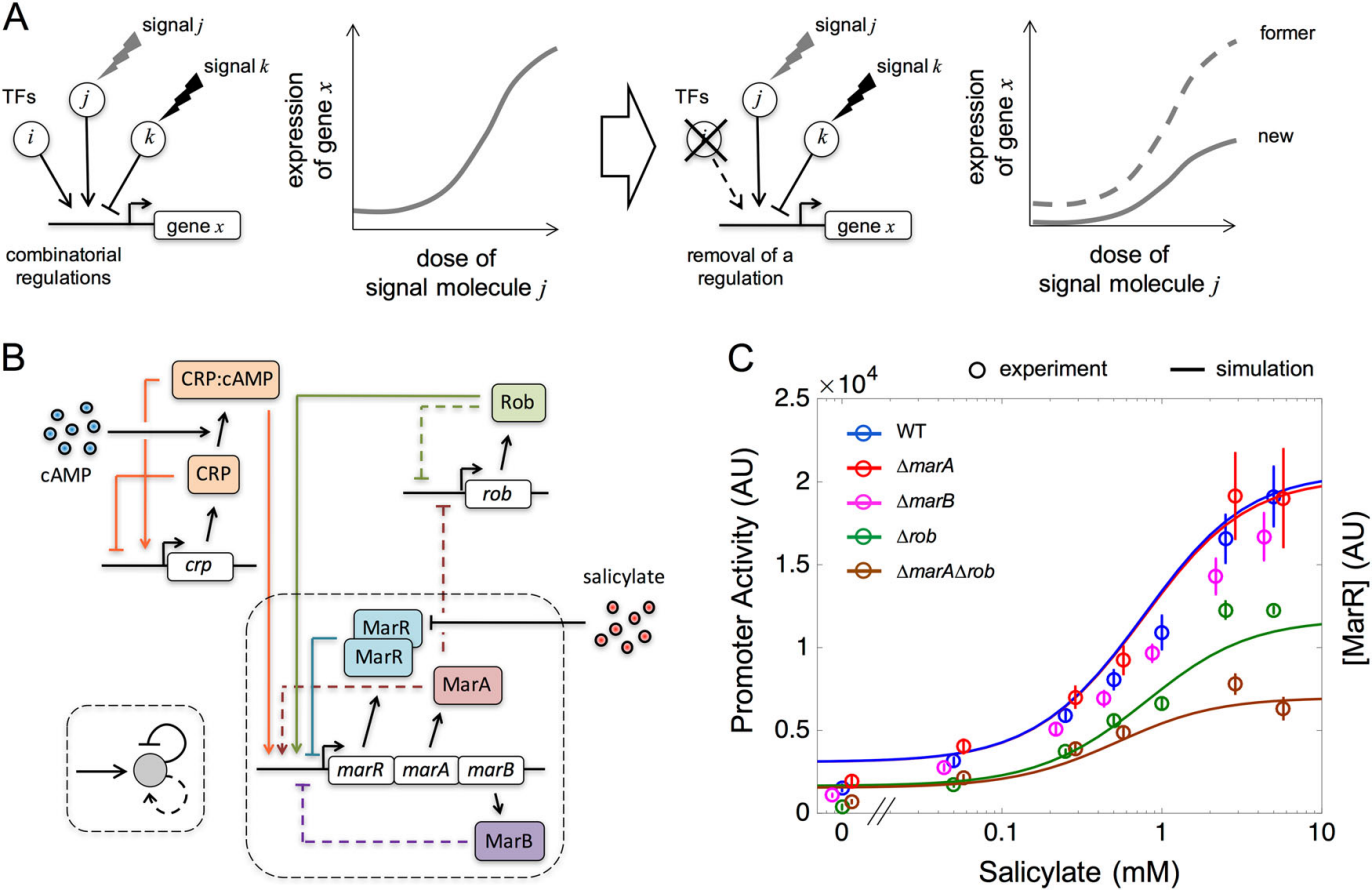
Inferring the regulatory complexity of the *mar* operon though the characterization of the input function. **a** A complex regulatory scheme can be associated to a particular input function (left) whose change when a regulator is perturbed helps us appreciate the role of this regulator (right). **b** Scheme of the *mar* core control network, which includes the components of the *mar* operon, MarR (a repressor acting as a dimer), MarA (an activator acting as a monomer), and MarB (a periplasmatic protein that may act as repressor), as well as two additional elements that are not part of the operon, CRP:cAMP and Rob (both monomeric activators). The *mar* operon (through MarA) controls the bacterial response to a number of toxic compounds, including antibiotics, and is also sensitive to metabolic signals (through CRP). Dashed lines indicate weak regulations. Inset illustrates the logical regulatory architecture of the operon. **c** Input function (promoter activity in steady state as a function of salicylate) measured by means of a YFP reporter system (YFP follows the dynamics of MarR^30^). Functions corresponding to the wild type and several mutant strains are shown. Open circles correspond to experimental data (error bars are standard deviations of three replicates); solid lines correspond to model predictions of gene expression levels (MarR, representation in arbitrary units, AU)

We propose here to utilize input functions in a complementary manner, by interpreting them as dissecting tools to reverse engineer the complexity of combinatorial regulation. How these functions change as we disturb the activity of the associated regulators would uncover the underlying principles behind a particular regulatory logic (Fig. 1a).^18^ To demonstrate this view, we examined the multiple antibiotic resistance (*mar*) response in *Escherichia coli* as a model system of complex regulation.^19^ The *mar* control system enables *E. coli* with intrinsic resistance, which makes it particularly attractive to explore, as well as the fact that homologous systems exist in other pathogenic bacteria.^20^ Specifically, the *cis*-regulatory region linked to the expression of the core agents of the response, the genes *marR*, *marA* and *marB*, combines several signals that correspond to global (CRP) and local (MarA, MarR, Rob) transcription factors.^21^ We introduced a mathematical model that integrates the known molecular details of the response to calculate the input functions with respect to its associated signals. Changes on the input functions as a consequence of certain mutations help explain the role of the regulators involved, and the rationale behind many of the properties of the system. Notably, the dual autoregulation being part of this combinatorial control turns out to be a core regulatory architecture with implications for the specificity and dynamics of the response.

## Results

### A bottom-up model predicts the input function of the *mar* operon with respect to salicylate

A set of regulators controls the expression of the *mar* operon (Fig. 1b). Within this set, one finds the three that constitute the operon itself: MarR, MarA, and MarB. MarR is the repressor of the system,^22^ MarA is the activator,^23,24^ and the third element, MarB, also seems to repress expression, but in an indirect manner.^25^ The system is additionally influenced by specific (Rob) and global (CRP) transcription factors, both presenting operator sequences in the *cis*-regulatory region^21^ (Supplementary Fig. S1A explicitly shows this region).

We integrated this information in a quantitative model of the response to anticipate the shape of the input function of the wild-type (WT), and several relevant mutants, as function of salicylate (salicylate inactivates the action of MarR^26^). The model incorporates known features like the strong repression of MarR,^22^ the weak action of MarA,^23,24^ and the independent binding between MarA and MarR, but see ref. 23. It also included competitive binding among MarA, Rob and CRP:cAMP (details and model parameters in Methods, Supplementary Information, and Supplementary Table S1).^27–30^ The theoretical results were combined with experimental measurements of promoter activity with the use of a YFP reporter under the control of the *marRAB* promoter^30^ (in the presence of glucose, so that the action of CRP:cAMP is inactivated;^31^ Methods).

Figure 1c shows the corresponding input functions (see also Supplementary Fig. S1B; MarB marginal effect was evaluated experimentally, Supplementary Fig. S2, but not included in the model). Experimental input functions corroborated theoretical predictions (*R* ^2^ = 0.927, by considering all 33 data points in Fig. 1c, Student’s *t*-test, d.f. = 25, *P* < 0.001). The validation of the theory confirmed in this way the model assumptions, and emphasized both the relevance of Rob, and the important interplay between MarA and Rob in the generation of the *mar* response.

### Rob scales up the *mar* input function

**Fig. 2:**
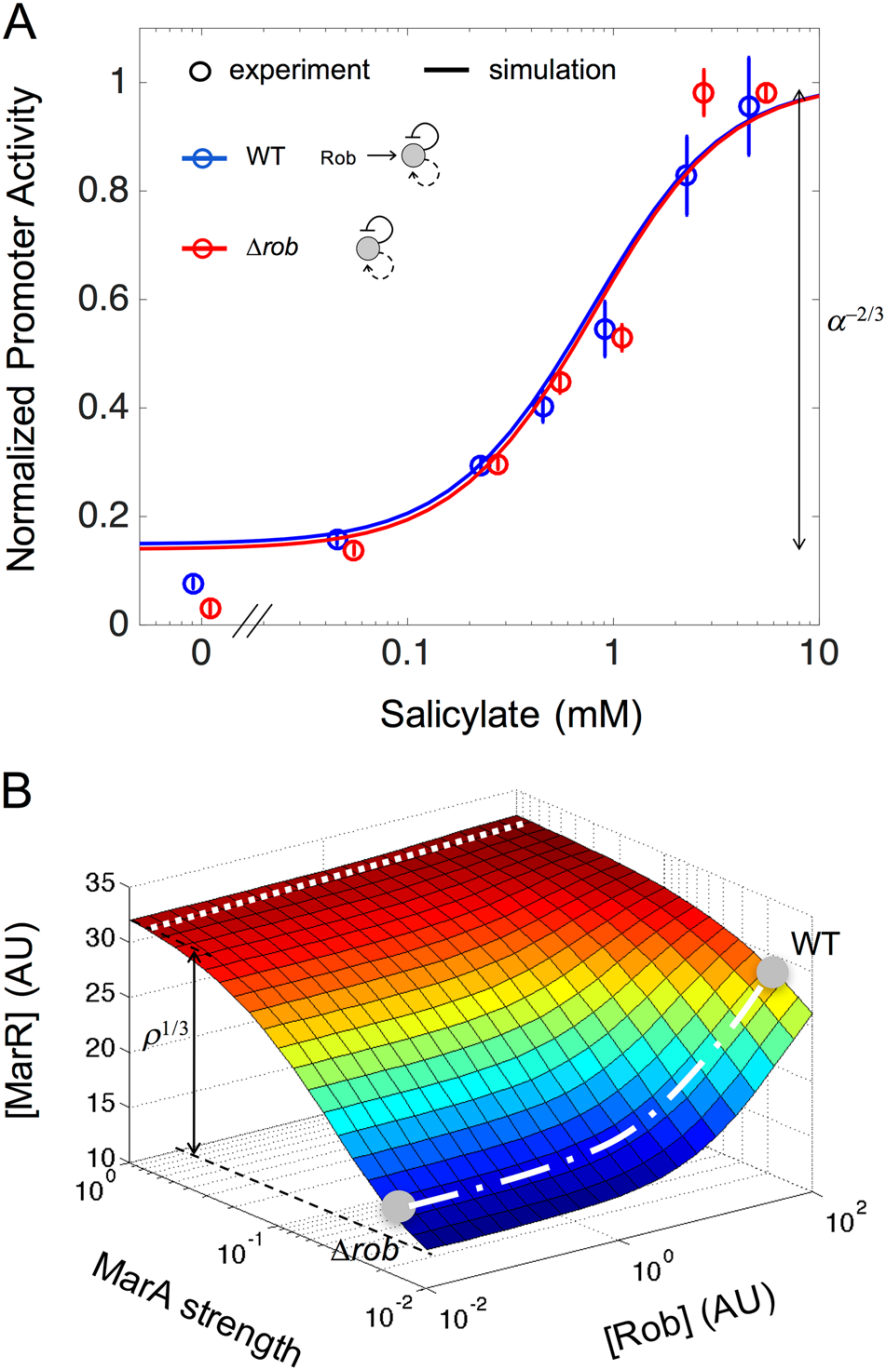
Effect of Rob on the *mar* operon input function. **a** Normalized input function (promoter activity in steady state relative to the maximum) as a function of the salicylate concentration for the wild-type (WT, blue) and ∆*rob* (red) strains. Open circles correspond to experimental data (error bars are standard deviations of three replicates), with input and output ranges of *R*_in_ = 31.11 ± 2.46 and *R*_out_ = 8.85 ± 0.04 (WT), and *R*_in_ = 20.88 ± 1.17 and *R*_out_ = 11.48 ± 1.03 (∆*rob*). Solid lines correspond to model predictions, with *R*_in_ = 28.32 and *R*_out_ = 6.77 (WT), and *R*_in_ = 27.51 and *R*_out_ = 7.17 (∆*rob*). Response fold change dependence on the fraction of functional MarR upon full induction (*α* parameter in the model, *α* ≈ 5%, Supplementary Table S1) is well predicted by the theory to be *R*_out_ ≈ *α* ^−2/3^ ≈ 7.4. **b** Both MarA activation strength and Rob concentration modulate the expression level of MarR in equilibrium (simulations for 5 mM salicylate). The effect or Rob becomes evident in a weak regime of MarA (natural system; dashed-dotted white line). The same scaling (≈ *ρ* ^1/3^) could be obtained if the action of MarA were strong, which would inactivate the effect of Rob (dotted white line). See text for details

Beyond the previous characterization, the regulatory role of Rob in the *mar* system remains, nonetheless, uncertain.^32^ The input function of a Δ*rob* strain (Fig. 1c) appeared qualitatively different to that of a WT strain; so we decided to examine these curves quantitatively. We used two complementary scores, the input (*R*_in_) and output (*R*_out_) dynamic range, which account for the “steepness” of the expression of the operon with respect to the dosage of the input signal (salicylate; the smaller the *R*_in_, the larger the ultrasensitivity), and its response fold change, respectively.^33^ For the WT strain (i.e., including Rob), we found *R*_in_ ≈ 31 and *R*_out_ ≈ 9, while in case of a Δ*rob* strain we obtained *R*_in_ ≈ 21 and *R*_out_ ≈ 11 (Methods). The relative similarity of these values is confirmed by a model simulation that revealed equivalent numbers (*R*_in_ ≈ 28 and *R*_out_ ≈ 7 for the WT and Δ*rob* systems), and agrees with previous experimental estimations of the activity of the regulators of the system.^22–25^ Presence, or absence, of Rob does not alter then the shape of the input function. This is clearly seen in Fig. 2a in which a matching between the functions of the WT and Δ*rob* systems is indeed observed after normalization (data were normalized by the maximum).

To further inspect the input function and the action of Rob, we obtained an analytical prediction of the factors influencing fold change, *R*_out_, by approximating the equilibrium expression of MarR with the mathematical model (Methods and Supplementary Information). The analysis shows that fold change depends only on the fraction of functional MarR (irrespective of Rob or MarA) as *R*_out_ ≈ *α* ^−2/3^, where *α* is the fraction of active MarR in conditions of saturating salicylate (*α* = 0.05, Supplementary Table S1; more details in the following sections). This prediction accurately fits the experimental data (Fig. 2a).

The analytical predictions also validated that the only effect of Rob is to scale the input function (by a factor of *ρ* ^1/3^; *ρ* is the activation fold, Methods and Supplementary Information). Note here that it is the weak activation of MarA what makes Rob a modulator of the expression of the operon. Figure 2b illustrates this conclusion by plotting the equilibrium concentration of MarR, as predicted by the model, in different scenarios characterized by MarA strength and Rob dosage (considering a regime of full induction, 5 mM salicylate). The dashed-dotted white line highlights a weak MarA condition (WT system) with different dosage of Rob (the limit of very low dosage represents a Δ*rob* system). Rob clearly scales the equilibrium. A modified model that featured instead a strong action of MarA does not show the effect (dotted white line, Fig. 2b). In the latter condition, the operon would additionally present high input dynamic range.

It is also noteworthy how the sensor of the system (MarR) is encoded in the same operon than the actuator MarA (see the early discussions on the importance of autogenous versus non-autogenous control^34^). We examined the consequences of this genetic architecture by contrasting the natural system with a hypothetical one in which the repressor is encoded in a different transcriptional unit. A model describing this variant system predicts a lower input dynamic range (*R*_in_ = 9) than the natural one (*R*_in_ ≈ 28, model prediction as well). This agrees with earlier work, which associates the occurrence of negative autoregulation to more linear input function curves, i.e., lower steepness of the response.^35,36^

In sum, we find that the negative autoregulation is the dominant feedback determining the main attributes of the input function, while Rob is a specific element that scales up expression levels (maintaining both input and output dynamic range, Fig. 2b). Moreover, scaling suggests that the regulation of Rob expression is not interfered by independent signals, which are not acting on the control of the core *marRAB* operon.

### Two-dimensional input functions uncover how Rob buffers the potential cross talk with cAMP signaling

**Fig. 3:**
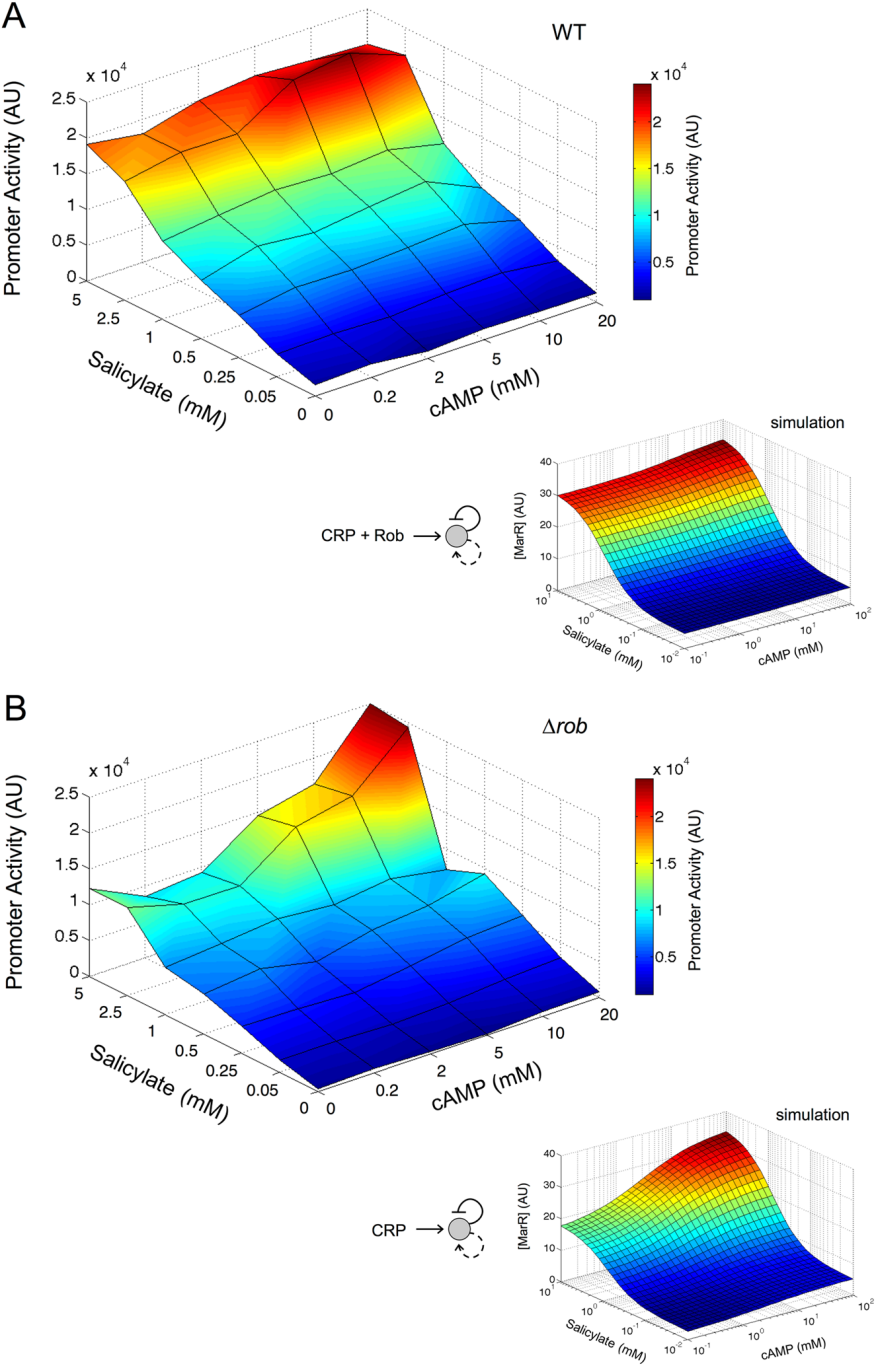
Input function of the *mar* operon with respect to salicylate and cAMP. Two-dimensional input function (promoter activity in steady state) as function of salicylate and cAMP (42 concentration combinations of the two input signals). **a** Wild-type system. **b** ∆*rob* system. Inset figures show model simulations of gene expression levels (MarR) for the corresponding system that anticipate the experimental data. Absence of Rob leads to the appearance of cross talk between signals

Global regulators beyond the specific (local) regulators of the *mar* system can additionally influence its expression^14^ (Fig. 1b). In particular, a CRP binding sequence overlapping with the *marA* operator (Supplementary Fig. S1A) makes the operon potentially responsive to metabolic stresses. To examine this possibility, we first measured a two-dimensional input function with respect to extracellular salicylate and cAMP signals in the WT strain (Fig. 3a; see Supplementary Fig. S3a for the corresponding temporal dynamics). Note that only extracellular cAMP binds to CRP to activate the operon in the presence of glucose.^11,31,37^ Remarkably, the response appeared nearly independent of cAMP.

We hypothesized that the lack of response could be linked to a second positive regulation that maintains high occupancy at the corresponding promoter region (assuming that MarA, Rob and CRP:cAMP compete for binding on the promoter). Because the activation by MarA is weak, we considered Rob as potential underlying element. Simulations of two-dimensional input functions with and without Rob supported the assumption (insets in Fig. 3a, b, respectively). To confirm this calculation, we obtained the experimental two-dimensional input function of the Δ*rob* strain. We observed a sizeable influence of cAMP as predicted (Fig. 3b; temporal dynamics in Supplementary Fig. S3B). Overall, we conclude that the increase in promoter activity due to Rob isolates the system from the potential interference of global signals.

### Derepression of the mar operon is limited by intracellular copper signaling

**Fig. 4:**
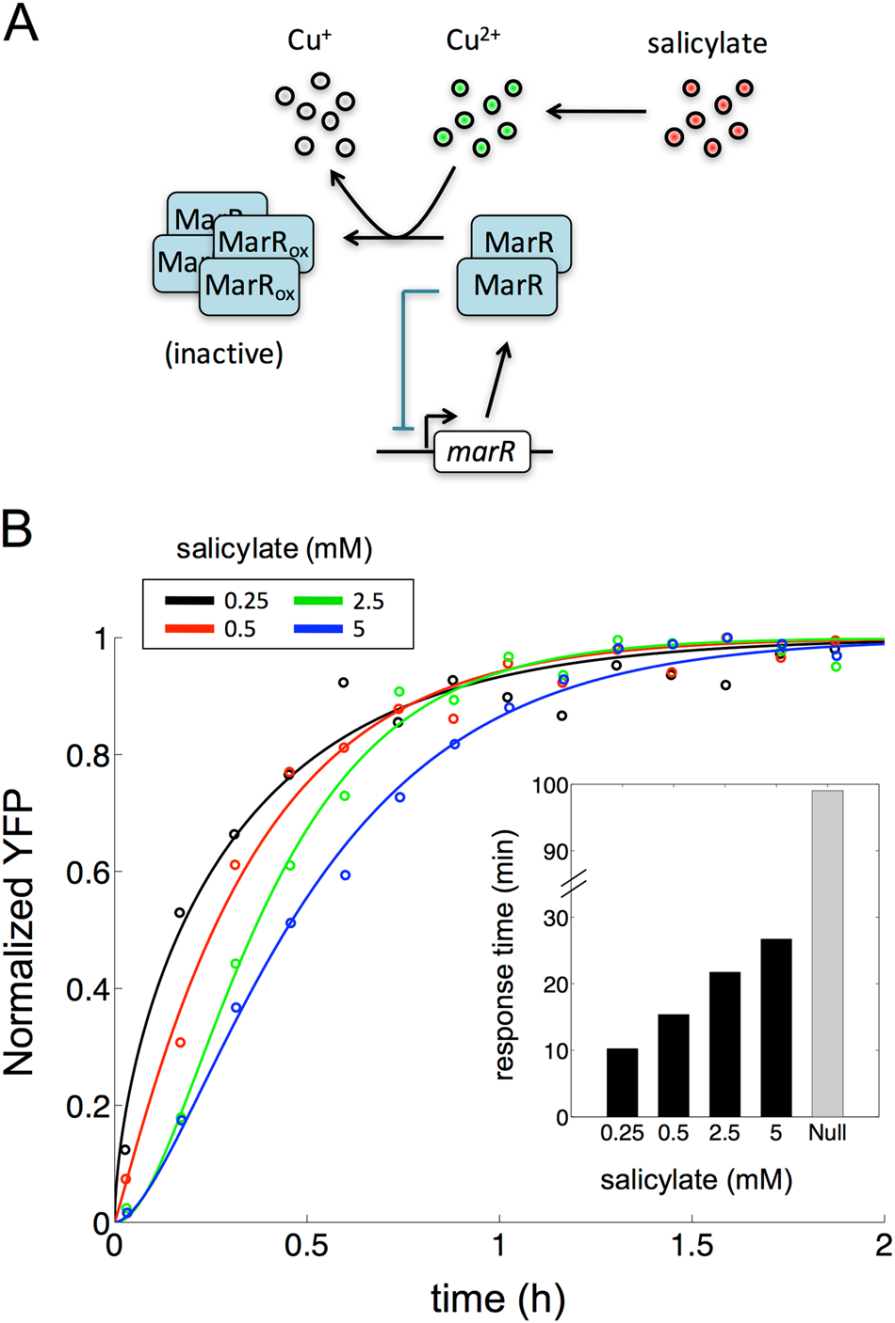
The effect of MarR as a sensor in the *mar* operon. **a** Scheme of the role of copper signaling in MarR regulation. Salicylate induces the intracellular accumulation of Cu^2+^ ions, which in turn oxidize MarR molecules. While non-oxidized MarR molecules form dimers (with repressor action), oxidized MarR (MarR_ox_) molecules form tetramers (inactive). **b** Temporal dynamics of the system, as given by the normalized fluorescence (YFP, relative to the maximum), for different salicylate concentrations. Small circles represent experimental data (averages of three replicates), solid lines represent fittings to an exponential model (data for wild-type strain). Inset shows the associated times to reach to half the steady state values (response times; gray bar corresponds to cell-cycle time, i.e., a null scenario in which the operon is considered to be constitutively expressed.^30^)

That salicylate releases the repression of the operon by the inactivation of MarR was a result discussed for two decades^26^ but the precise mechanism of which was unknown. However, recent experimental work indicated that salicylate induces the accumulation of Cu^2+^ ions within the cell what eventually oxidizes MarR and inhibits its action^38^ (Fig. 4a). As the accumulation of copper is highly toxic for bacteria (levels could reach at most 10 μM since cells die otherwise^39^), this could represent a limitation in the oxidation of MarR proteins and thus in the full transcription of the operon. We examine this potential restriction next.

At maximal salicylate induction, one finds ~ 1000 molecules of MarR per cell.^40^ Using this value and the oxidation curve of MarR by Cu^2+^ (Supplementary Fig. S4A),^14^ we found that around 50 molecules could remain non-oxidized, even with maximal induction (what fixes to 5% the value of the partial derepression parameter *α* mentioned in an earlier section, Supplementary Table S1). A certain amount of repression could still be active in this regime, given that few molecules of MarR inactivate operon expression (binding constant of MarR is of the order of nM^22^). Consequently, a higher number of molecules of MarR at maximal induction would further hinder activation of the response. We propose that the inefficient translation rate of MarR^40^ might be beneficial to successfully release repression by reducing MarR concentration, given the restrictions of copper signaling.

The residual presence of a negative autoregulation can influence other aspects. In particular, it brings about a faster rise-time from the uninduced to the induced steady state.^41,42^ To evaluate this effect, we mimicked the strength of the remaining repression with the use of different salicylate doses. As predicted, we found that the lower the dose (stronger “residual” autoregulation) the faster the dynamics (Fig. 4b). Moreover, the response time of a fully derepressed system –maximal salicylate– should be that of a constitutively expressed stable protein, i.e., the cell cycle.^41^ We found though a shorter response time what appears to confirm that at saturating salicylate dose a remnant amount of active repression is present (speedup due to negative autoregulation is partially effective). Simulations performed with the mathematical model verified the experimental data on dynamics (Supplementary Fig. S4B), and underlined how the specific value of this remnant repression determines the input function (Supplementary Fig. S4C).

### The *mar* operon presents no memory

**Fig. 5:**
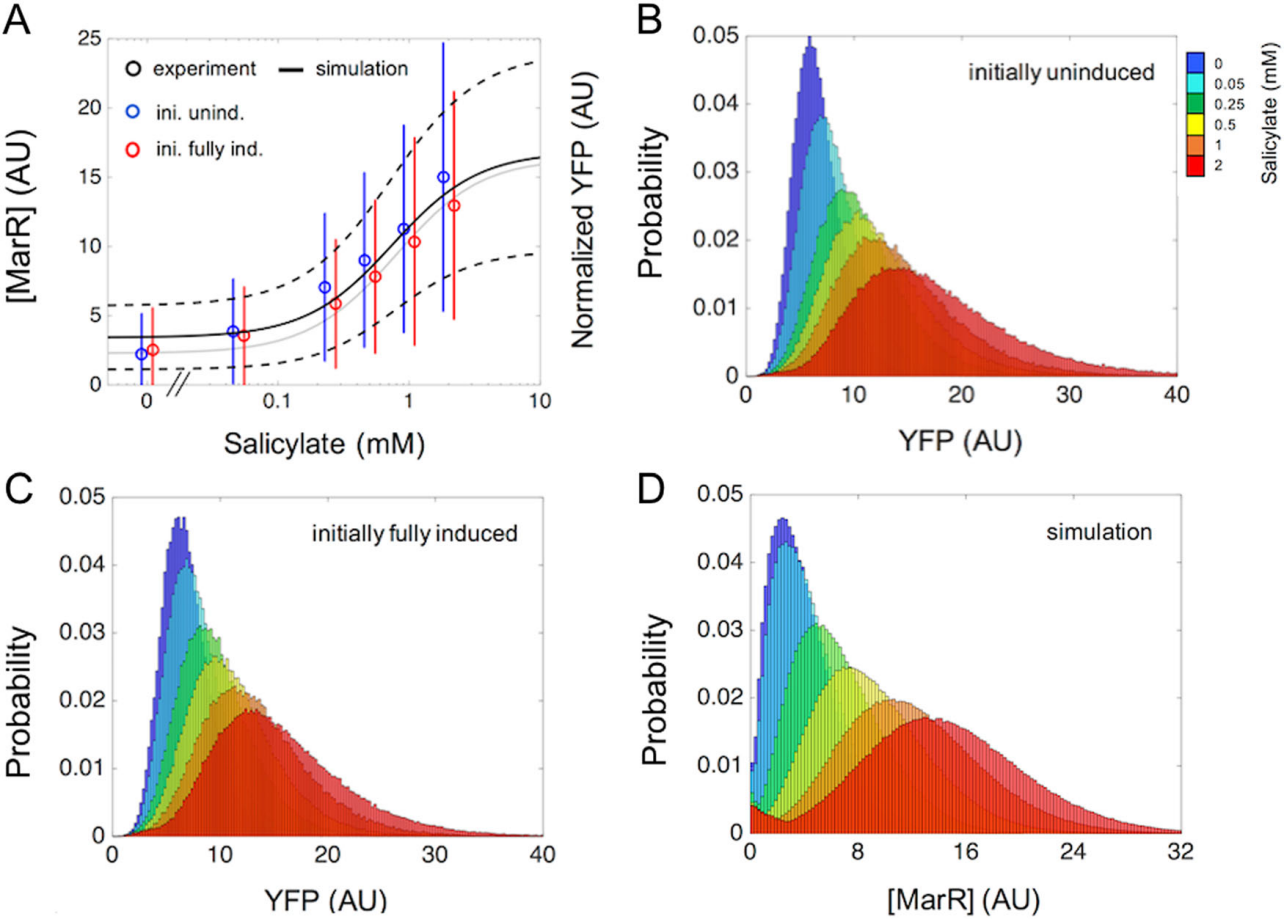
Single-cell response of the *mar* operon with two unrelated previous exposures to inducer. **a** Open circles denote the median respond to salicylate corresponding to single-cell experimental data using the YFP reporter system (error bars are standard deviations; representation in arbitrary units AU). Bacteria were initially uninduced (blue) or fully induced (red) before exposure to the different dosages of salicylate. Solid gray line indicates the deterministic input function obtained by simulation, while the solid black line represents the median input function predicted by a stochastic model (dashed lines represent standard deviations over the median; all data and simulations here for a ∆*rob* system). **b** Probability distributions in steady state for different salicylate concentrations for cells previously uninduced. **c** As before when cells were previously fully induced (5 mM salicylate). **d** Model simulation of the probability distributions in steady state for different salicylate concentrations were also independent of the presence/absence of a preceding induction (no hysteresis)

By using the input function as quantitative tool, we can examine whether the *mar* operon exhibits hysteresis^43^: a differential response depending on its previous exposure to salicylate. Hysteresis is usually associated to bistable expression, which is necessarily observed by monitoring individual cells. Therefore, we quantified the induction of the *mar* operon at the single-cell level for two initial conditions in which the bacterium has, or has not, previously experienced salicylate. Figure 5a shows data corresponding to median and variance of the single-cell behavior of the population for each dosage level (Methods). Median values outline a median input function, while the variance reflects the variability in the individual response.^43^ A similar curve independent on the previous exposure is observed, which approximately follows the simulation of the input function with the deterministic model (Fig. 5a, shown in gray for comparison).

To further inspect any possible presence of bistability, we showed the corresponding distributions of the single-cell expression of YFP in a population (YFP follows the dynamics of MarR^30^) (Fig. 5b, c for the initially uninduced or induced condition, respectively; results for Δ*rob* strain). The similarity between distributions corroborates the lack of memory, an effect similarly obtained in a WT strain (Supplementary Fig. S5, we considered above a Δ*rob* strain to focus on the core operon). This behavior is correctly described with a stochastic version of the model (Fig. 5a black lines, and Fig. 5d) (i.e., noise does not originate memory^44^).

### A different genetic implementation of the *mar* dual autoregulatory logic would change the input function

**Fig. 6:**
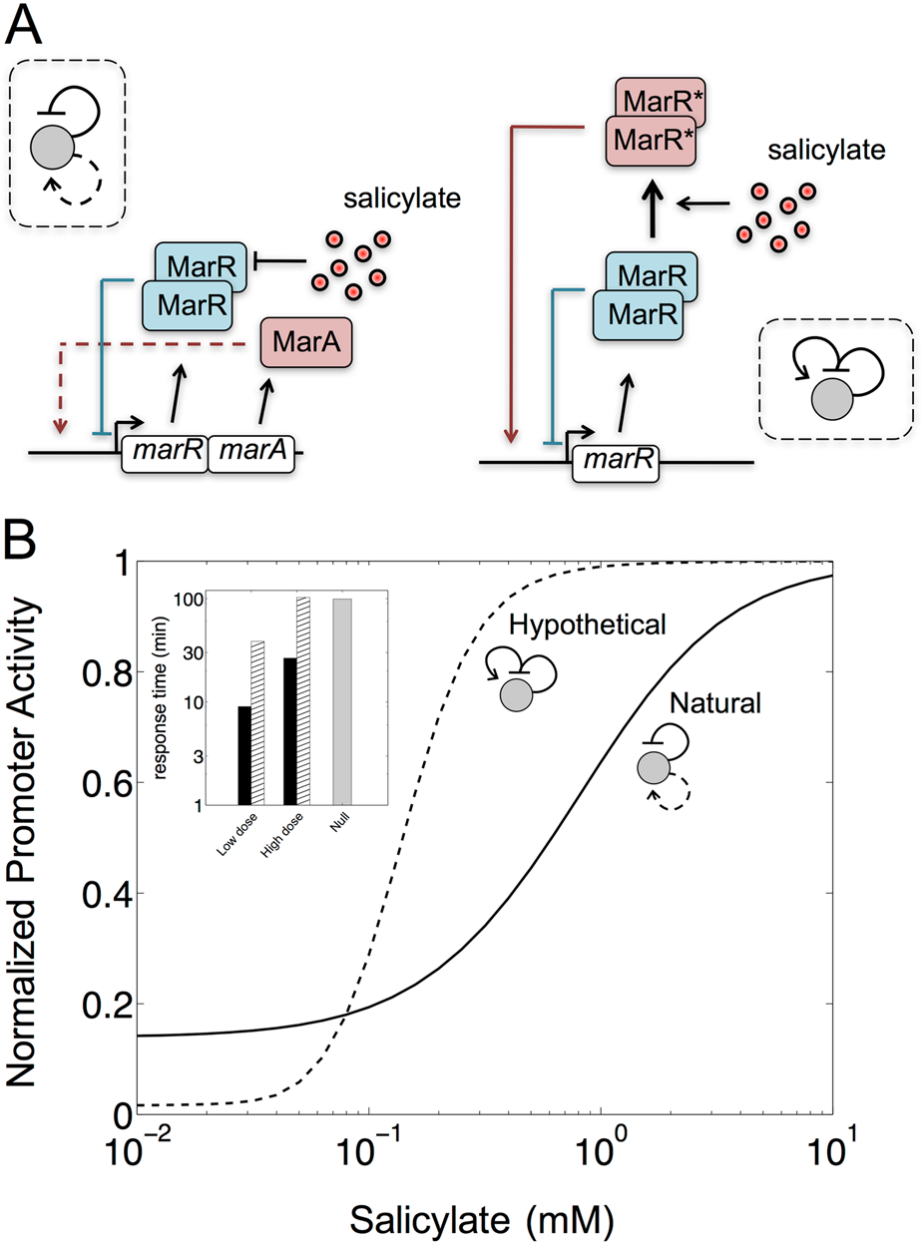
Predicted input function of an alternative genetic implementation of the *mar* dual autoregulatory motif. **a** Schemes of two possible genetic implementations of the *mar* core network with positive and negative autoregulation (in the absence of Rob and CRP:cAMP).^58^ Left scheme correspond to the natural circuit. Right scheme corresponds to the hypothetical circuit where the oxidized MarR works as an activator (with competitive binding). **b** Input function (model simulations of normalized promoter activity relative to the maximum) with respect to salicylate. Solid line denotes the natural circuit (input and output range being *R*_in_ = 27.51 and *R*_out_ = 7.17, respectively). Dashed line denotes the hypothetical one (*R*_in_ = 5.16 and *R*_out_ = 62.17). Inset shows the associated response times (time to reach steady state) of these two implementations. Black bars correspond to the natural circuit. Hatched bars to the hypothetical circuit (gray bar shows a null reference value relative to a system that would start the production of the *mar* operon at a constant rate). Low dose corresponds to 0.01 mM and high dose to 10 mM

Finally, we noted that the bicistronic two-component architecture of the *mar* circuit is unique in *E. coli*, while its other three responses exhibiting dual autoregulation correspond to bifunctional regulators (Supplementary Table S2). For instance, when cAMP is present in the medium, CRP works as an activator (complex CRP:cAMP), but it functions as repressor otherwise **(**Fig. 1b).^31^ We thus asked how genetic implementation could matter for the characteristics of the *mar* input function, as distinct implementations led to functional consequences in other studies.^42,45,46^ A hypothetical architecture in which the oxidized MarR (due to salicylate) switches from repressor to activator action was considered (Fig. 6a); we also assumed parsimoniously that the regulator works as a dimer in both cases, and that they bind competitively.

We compared the input functions by carrying out a mathematically controlled comparison between the two designs with as many similar parameters as possible^34^ (Methods; this assumes similar binding affinities of MarR and oxidized MarR what can be relaxed, Supplementary Fig. S6). The WT circuit showed a larger input dynamic range (approximately 5-fold, Fig. 6b). In contrast, the hypothetical one exhibited much higher fold change (about 9-fold to the WT architecture, Fig. 6b). One could speculate then that a bicistronic implementation preferentially evolved to sense a larger input range of signal molecule concentrations, while maintaining moderate fold change. Alternatively, responses governed by means of bifunctional regulators could require either more fold change to fine-tune very large regulons (this could apply for instance to CRP^47^), or higher ultrasensitivity, i.e., more nonlinearity, to trigger the response under a very narrow signal range (this could be relevant to ChbR, which activates the expression of the *chb* operon only when sufficient flux through their associated pathway is sensed^48^). In addition, the rise-time to reach the induced steady state was faster in the natural circuit (inset in Fig. 6b; see also Supplementary Figs. S6–7). More in detail, we found that the system with a bifunctional regulator responded similarly to a circuit under simple regulation at high salicylate doses (the regulator would dominantly function as activator), but exhibited speedup at very low doses. In the latter situation, there would be a combined action of positive and negative regulation over the promoter, similar to the one governing the induction of the natural *mar* circuit.

The cost of encoding a bicistron –two genes to express instead of one in the bifunctional scenario– could hence be counteracted by certain functional attributes that provide an advantage in environments in which response speed and large input dynamic range become beneficial traits (note that some attributes remain comparable for *monomeric* bifunctional regulators, Supplementary Fig. S6).

## Discussion

Here, we put forward the use of the input function of a response system as a quantitative tool to deconstruct its regulation. We validate this approach by examining the *cis*-regulation of the *mar* response in *E. coli*, as a model of complex expression control in prokaryotes.^19^ The analysis of the input function (in wild-type and several regulator-deleted strains) helps us unravel the role of different regulators on the transcription rate of the *marRAB* operon, and underlines the implications of its dual autoregulatory architecture.^30,49^ The function is well predicted by a mathematical model that assumes independent binding between the activator (MarA) and repressor of the system (MarR). Investigation of the regulatory role of one of the additional regulations, i.e., Rob, reveals two important features: (1) Rob scales the expression of the system, a result that is made possible by the weak strength of the actuator protein MarA, and (2) the strengthening of the response by Rob also sets apart the operon from metabolic signals coupled to CRP, other of the transcription factors involved. The activation by CRP might consequently be interpreted, in this *cis*-regulatory context, as an evolutionary contingency related to a more general program of regulation.^14^

Moreover, copper signaling has been recently documented as the mechanism connecting the signal (salicylate) to the sensor element (MarR). The accumulation of intracellular Cu^2+^ in response to salicylate oxidizes MarR preventing its binding to DNA.^38^ Because the intracellular concentration of this cation is bounded (to avoid toxicity), copper balance imposes maximal repressor abundance for which the promoter can be derepressed at saturating signal levels, a constraint providing a rationale for the limited translation rate observed for MarR.^40^

The weak strength of the positive autoregulation (through MarA) also causes the absence of memory in the system, a property that one expects since the transcriptional circuit includes a positive feedback.^43^ Regulatory logic does not necessarily determine function and the biochemical parameters matter, as we find in this example. The particular genetic implementation of the logic also matters. We identified alternative designs of the dual autogenous control in *E. coli* that consists on dual regulators rather than polycistrons (carrying both repressor and activator). Dual regulators switch between repression and activation due to the presence of its cognate inducer (CRP, for instance, works in this way^31^). Which are the differences between these two implementations? To explore this question, we apply the input function again as a quantitative tool to examine a hypothetical *mar* system in which oxidized MarR turns into an activator (by means of a mathematically controlled comparison^34^). The more compact genetic design exhibits a smaller input range (i.e., the reaction to a signal gradient is more sigmoidal), a wider output range (what enables, for example, to regulate differentially large regulons as it could be the case of CRP), and a slower response (with no speedup at high dosages). The result exemplifies how the response associated to a given regulatory logic is certainly influenced by its genetic design.^42,45,46^

More broadly, this work supports a view of the input function of a gene as a dissecting tool that can make clear the logic of its regulation.^18,37^ We identified an underlying principle in which the presence of dual autoregulation in a complex transcriptional control system can very much delineate the shape of this function. An additional positive regulation limits the interference of other general signals on the promoter by scaling the response. Our study contributes in this way to the glossary of regulatory structures and their input functions that needs to be quantified in both prokaryotic and eukaryotic systems.

## Methods

### Strains, culture media and reagents

A strain called IE01 carrying two fluorescent reporters was engineered to measure the activity of the WT *mar* core network. It is based on *E. coli* K-12 MG1655. The strain contains a chromosomal copy of the gene coding for a yellow fluorescent protein (YFP) under the control of the *marRAB* promoter, and the gene coding for a cyan fluorescent protein (CFP) expressed with a constitutive promoter as a control.^50^ Four more strains called TC01, DB01, IE02, and TC02 were constructed by deleting fundamental genes of the system, with the application of the knockout protocol on the strain IE01.^51^ TC01 is a ∆*marA* strain, DB01 a ∆*marB* strain, IE02 a ∆*rob* strain, and TC02 a ∆*marA*∆*rob* strain.

Medium LB was always used for overnight cultures. Minimal medium M9 (1x M9 salts, 2 mM MgSO_4_, 0.1 mM CaCl_2_, 0.4% glucose, 0.05% casamino acids, 0.05% vitamin B1) was used to grow cells during characterization experiments. To induce the *marRAB* promoter, different concentrations of salicylate and cAMP (Sigma Aldrich) were used. Note that glucose inhibits the production of internal cAMP.

### Fluorometry

Cultures inoculated from single colonies (2 ml, three replicates; strains IE01, TC01, DB01, IE02, and TC02) were grown overnight in LB medium supplemented with 0.4% glucose at 37 °C and 170 rpm. Cultures were then diluted 1:200 in M9 minimal medium, and were grown for 2 h at the same conditions. Cultures were then used to load the wells of the microplate (0.2 mL, Thermo Scientific) with salicylate and cAMP when appropriate. The microplate was assayed in a fluorometer Victor × 2 (Perkin Elmer) measuring absorbance (600 nm), yellow fluorescence (497 nm, 535 nm), and cyan fluorescence (434 nm, 479 nm) for 4 h at 37 °C with shaking.

In case of pre-induction, cultures inoculated from single colonies (2 ml, three replicates; strain IE01) were grown overnight in LB medium supplemented with 0.4% glucose and 5 mM salicylate at 37 °C and 170 rpm. Cultures were then diluted 1:200 in M9 minimal medium with 5 mM salicylate, and were grown for 5 h at the same conditions. Cells were pelleted by centrifuging 10 min at 13,000 rpm, and resuspended in fresh M9 minimal medium. Cultures were then used to load the microplate as described above.

The normalized fluorescence of YFP was calculated as the ratio of fluorescence and absorbance, having subtracted background values. The growth rate of cells was calculated as the slope of the linear regression between the log of absorbance and time. Promoter activity in steady state was calculated by multiplying the normalized fluorescence and the growth rate.^52^ Data were analyzed with Matlab (MathWorks). The means and standard deviations of three replicates were computed. See Supplementary Information for further details.

### Flow cytometry

Cultures inoculated from single colonies (2 ml; strains IE01 and IE02) were grown overnight in LB medium with and without salicylate (5 mM) at 37 °C and 170 rpm (to have uninduced and pre-induced cultures). Cultures were subsequently diluted 1:200 in M9 minimal medium with different concentrations of salicylate, and were grown for 4 h at the same conditions. Cultures were then transferred to Eppendorf tubes (1 ml). The fluorescence distribution of each sample was obtained with a flow cytometer MACSQuant VYB (Miltenyi Biotec) measuring yellow fluorescence (488 nm, 525 nm). Using delimited forward and side scatter ranges with the commercial software installed in the machine gated events. To calculate the normalized fluorescence, the background value was subtracted, and the resulting values were rescaled according to the mathematical model. The medians and standard deviations of the distributions were computed.

### Quantitative PCR (qPCR)

Cultures inoculated from single colonies (2 ml, three replicates; strains IE01 and DB01) were grown overnight in LB medium at 37 °C and 170 rpm. Cultures were then diluted 1:200 in M9 minimal medium, and were grown for 2 h at the same conditions. Cells were pelleted by centrifuging 2 min at 13,000 rpm, and resuspended in TE buffer (10 mM Tris-HCl, pH 8.0, 1 mM EDTA). Cells were broken with phenol-chloroform and vortexing thoroughly, recovering the RNA in the aqueous phase. Samples were then passed through a silica-based, DNA-clean column (Zymo), and were eluted in TE buffer with application of DNase I (Fermentas). Total RNA eluted was quantified in a NanoDrop.

One-step SYBR PrimerScript RT-PCR Kit II (Takara) was used for detection, following the Kit protocol for preparing the reaction volumes. 16S ribosomal RNA (rRNA) was used as housekeeping gene to normalize RNA quantity in each reaction. The primer sequences for amplifying *marA* and 16S rRNA were taken from previous work.^53,54^ Reactions in triplicate were carried out using a Step One Plus Real-Time PCR System (Applied Biosystems). The thermal cycling program for amplification was 5 min at 42 °C, 10 s at 95 °C, and 40 cycles of 5 s at 95 °C and 34 s at 60 °C (Shuttle PCR), followed by default melting curves.

### Calculation of dynamic ranges

*R*_out_ is the ratio between the maximal and minimal promoter activities (i.e., fold change). *R*_in_ is the ratio between the salicylate doses at which the system shows 90% and 10% of its maximal activity (i.e., steepness of the response^33^). Fittings shown in Supplementary Fig. S1B were used to calculate *R*_out_ and *R*_in_ associated to experimental data (errors derived by bootstrapping). Model simulations were used to perform theoretical predictions of *R*_out_ and *R*_in_.

### Mathematical model

We briefly describe here the basic features of the model describing the dynamic response of the *mar* core network (see Supplementary Information for details). The model consists of two differential equations. MarA (*x*) and MarR (*y*) normalized concentrations act as model variables, while time is given as number of cell cycles (in our experimental conditions, cell cycle of 99 min for 5 mM salicylate). For simplicity, we did not consider the marginal regulatory effect of MarB (Supplementary Fig. S2). The model also includes as external variables the normalized concentrations of Rob (*z*) and cAMP (*c*). It reads *dx*/*dt* = *βP*_*mar*_ - *δx* and *dy*/*dt* = *P*_*mar*_ *−* *y*, where *P*_*mar*_ is promoter activity, *β* the ratio between the translation rates of MarA and MarR (*β* = 30, as MarR is inefficiently translated^40^), and *δ* the degradation rate of MarA (*δ* = 60, as MarA is efficiently degraded by Lon protease^55^). By knowing that the *marRAB* promoter is activated by MarA, Rob, and CRP:cAMP (acting as monomers), and repressed by MarR (acting as dimer), promoter activity reads *P*_*mar*_ = *P*_*0*_ [1 + *ρ* (*κx* + *κz* + *c*)] / [(1 + *κx* + *κz* + *c*) (1 + *y*_0_^2^)], where *P*_*0*_ is the basal production rate (*P*_*0*_ = 12), *ρ* the activation fold of the system (*ρ* = 10), and *κ* the ratio between the effective dissociation rates of MarR and MarA (*κ* = 0.02, as the repression is strong and the activation weak^22–24^). In the limit, we have *y*_0_^2^ » 1 and *κx* « 1, which served to solve the model analytically (see below). In steady state, the concentration of MarA is proportional to the concentration of MarR (*x*_∞_ = *βy*_∞_/*δ*).

Salicylate and other compounds, including antibiotics, induce the oxidation of MarR, what inhibits the repressor action (*y*_0_ represents the concentration of non-oxidized MarR^38^). Here, we wrote *y*_0_ = *y* [1 + *α* (*S*/*θ*)^*ν*^] / [1 + (*S*/*θ*)^*ν*^], where *α*, *θ* and *ν* are three parameters that model the action of salicylate (*S* denotes its concentration in mM; *α* = 0.05, *θ* = 0.13 mM, *ν* = 1.4, ref. 30); in particular, *α* denotes the remaining fraction of functional MarR upon full induction with salicylate.

The concentration of Rob was considered constant (*z* = 100), neglecting the influence of MarA.^24^ The normalized concentration of cAMP is *c* = *C*/*K*_*C*_, where *C* is the external cAMP concentration (in mM) and *K*_*C*_ = 5 mM. The model was adapted to account for the dynamics of other regulatory architectures. In case of ∆*marA*, we set *x* = 0; and in case of ∆*rob*, *z* = 0. Note that the model does not account for MarB, dynamics of both WT and ∆*marB* systems are consequently the same.

We can approximate the equilibrium expression of MarR to obtain analytical expressions (Supplementary Information). For the WT system, the solution reads as *y*_eq_ ≈ (*P*_*0*_ *ρ*)^1/3^ in the absence of salicylate, and *y*_eq_ ≈ (*P*_*0*_ *ρ*/*α* ^2^)^1/3^ in the presence of a saturating dose. And for the Δ*rob* system, it reads *y*_eq_ ≈ *P*_*0*_^1/3^ or *y*_eq_ ≈ (*P*_*0*_/*α* ^2^)^1/3^ in the absence or presence of salicylate, respectively.

### Stochastic simulation

A Langevin approach was followed to model the stochastic response of the system.^30,56^ For that, we considered a scenario of quasi-steady state (*dx*/*dt* = 0, as *δ* » 1). Then, on the *dy*/*dt* equation, a Gaussian white noise with constant amplitude (*q*) was added. Here, *q* ^2^ was assumed proportional to *y*_eq_ (*q* ^2^ = 0.11 *y*_eq_), the stationary solution of the system (*dy*_eq_/*dt* = 0). To solve this stochastic differential equation in steady state, we used the Fokker–Planck equation,^57^ leading to the probability distribution of *y*.

### Alternative mathematical model

To model the dynamic response of the hypothetical *mar* core network in which the oxidized MarR (*y* - *y*_0_ denotes its normalized concentration) acts as an activator, we just took one differential equation, *dy*/*dt* = *P*_*mar*_ *−y*. Now, promoter activity reads *P*_*mar*_ = *P*_*0*_ [1 + *ρ* (*y−y*_0_)^2^]/[1 + (*y−y*_0_)^2^ + *y*_0_^2^], since we considered competitive binding (Supplementary Information). The competitive binding assumption rests on the knowledge of the modular architecture of the proteins. In our hypothetical dual regulator MarR protein the domain of DNA binding would be maintained, while the ligand could be modifying the domain of the interaction with the polymerase. More generally, in order to quantitatively compare the functional features of the wild-type and alternative designs, we applied Savageau’s mathematically controlled comparison approach, in which the parameters of the two systems are as similar as possible.^34^ Further biochemical assumptions, like the asymmetry of binding affinities, were also examined in Supplementary Fig. S6.

### Data availability

Supplementary information includes a more detailed description of experimental methods, models and analytical solutions, seven figures and two tables. Further data is available upon request.

## Electronic supplementary material

Supplementary Table S1

Supplementary Table S2

Supplementary

Supplementary Figure S1

Supplementary Figure S2

Supplementary Figure S3

Supplementary Figure S4

Supplementary Figure S5

Supplementary Figure S6

Supplementary Figure S7
