# Supplementary material for: Deconstructing a multiple antibiotic resistance regulation through the quantification of its input function
Source: NPJ Syst Biol Appl. 2017 Oct 6;3:30. doi: 10.1038/s41540-017-0031-2 (PMC5630622; doi:10.1038/s41540-017-0031-2)
Supplement: Supplementary file 1 — Supplementary Table S1 [file 41540_2017_31_MOESM1_ESM.docx]

**Table S1:** Nominal values of the parameters used in the model.

| Parameter | Value | Note | Reference |
| --- | --- | --- | --- |
| *β* | 30 | _With RBS calculator_ | Salis *et al*., 2009 |
| *δ* | 60 | Protein half-life of ~1 min, and cell cycle of ~100 min. | Griffith *et al*., 2004 |
| *P*_0_ | 12 | Assuming a production rate of ~1 nM/min, and a cell cycle of ~100 min. | Levine *et al*., 2007 |
| *ρ* | 10 | Experimental data show ~4-fold increase in expression due to the direct effect of MarA. | Martin et al., 1996 |
| *κ* | 0.02 | MarA-DNA dissociation constant 50 times larger than the MarR-DNA dissociation constant. | Rodrigo *et al.*, 2016 |
| *θ* | 0.13 mM | Adjusted to get the experimental transfer function in the reference. | Cohen *et al*., 1993 |
| *ν* | 1.4 | Adjusted to get the experimental transfer function in the reference. | Rodrigo *et al.*, 2016 |
| *α* | 0.05 | Assuming [Cu^2+^] / [MarR] ≈ 5 upon induction with 5 mM salicylate | Hao *et al.*, 2014 |
| *K_C_* | 5 mM | Adjusted to get the experimental transfer function in the reference. | Setty *et al*., 2003 |

Salis, H.M., Mirsky, E.A. & Voigt, C.A. Automated design of synthetic ribosome binding sites to control protein expression. *Nat. Biotechnol.* **27**, 946-950 (2009).

Griffith, K.L., Shah, I.M. & Wolf, R.E. Proteolytic degradation of Escherichia coli transcription activators SoxS and MarA as the mechanism for reversing the induction of the superoxide (SoxRS) and multiple antibiotic resistance (Mar) regulons. *Mol. Microbiol.* **51**, 1801-1816 (2004).

Levine, E., Zhang, Z., Kuhlman, T. & Hwa, T. Quantitative characteristics of gene regulation by small RNA. *PLoS Biol.* **5**, e229 (2007).

Martin, R.G., Jair, K.W., Wolf Jr, R.E. & Rosner, J.L. Autoactivation of the marRAB multiple antibiotic resistance operon by the MarA transcriptional activator in Escherichia coli. *J. Bacteriol.* **178**, 2216-2223 (1996).

Cohen, S.P., Levy, S.B., Foulds, J. & Rosner, J.L. Salicylate induction of antibiotic resistance in Escherichia coli: activation of the mar operon and a mar-independent pathway. *J. Bacteriol.* ***175****, 7856-7862* (1993)*.*

Rodrigo, G., Bajic, D., Elola, I. & Poyatos, J.F. Antagonistic autoregulation speeds up a homogeneous response in Escherichia coli. *Sci. Rep.* **6**, 36196 (2016).

Hao, Z. et al. The multiple antibiotic resistance regulator MarR is a copper sensor in Escherichia coli. *Nat. Chem. Biol.* **10**, 21-28 (2014).

Setty, Y., Mayo, A.E., Surette, M.G. & Alon, U. Detailed map of a cis-regulatory input function. *Proc. Natl. Acad. Sci. USA* **100**, 7702-7707 (2003).
