# Supplementary material for: Deconstructing a multiple antibiotic resistance regulation through the quantification of its input function
Source: NPJ Syst Biol Appl. 2017 Oct 6;3:30. doi: 10.1038/s41540-017-0031-2 (PMC5630622; doi:10.1038/s41540-017-0031-2)
Supplement: Supplementary file 2 — Supplementary Table S2 [file 41540_2017_31_MOESM2_ESM.docx]

**Table S2:** *E. coli* operons with positive and negative autoregulation.

| Regulator^1,2^ | Operon | Description | Molecular implementation^3^ |
| --- | --- | --- | --- |
| ChbR | *chbBCARFG* | Repressor that becomes activator in response to chitobiose. AraC/XylS family of transcriptional regulators. | Multifunctional  regulator |
| CRP | *crp* | Repressor that becomes activator in response to cAMP. CRP-FNR superfamily of transcription factors. | Multifunctional  regulator |
| LldR | *lldPRD* | Repressor that becomes activator in response to lactate. GntR family of transcriptional regulators. | Multifunctional  regulator |
| MarR  MarA | *marRAB* | Control of multiple antibiotic resistance. AraC/XylS family of transcriptional regulators. | Distributed  regulation |

^1^ The operons with dual regulators (according to RegulonDB, ref. 59 in main text) *ada-alkB, araC, dnaAN-recF, gadAXW, leuO, melR, murR,* and *phoPQ* are not considered as systems with antagonistic autogenous control in the sense of this study.

^2^ AraC or MelR, both transcriptional regulators of the AraC/XylS family, repress their own expression whereas they exert dual regulation (with arabinose or melibiose as inducers) on their targets.

^3^ A *multifunctional regulator* is a transcription factor that works as a repressor and as an activator. *Distributed regulation* entails two independent transcription factors, a repressor and an activator.
