# Supplementary material for: Deconstructing a multiple antibiotic resistance regulation through the quantification of its input function
Source: NPJ Syst Biol Appl. 2017 Oct 6;3:30. doi: 10.1038/s41540-017-0031-2 (PMC5630622; doi:10.1038/s41540-017-0031-2)
Supplement: Supplementary file 3 — Supplementary [file 41540_2017_31_MOESM3_ESM.pdf]

Supplementary Information

Deconstructing a multiple antibiotic resistance regulation through the quantification of its input function

Guillermo Rodrigo[1], Djordje Bajić[2], Ignacio Elola[2], and Juan F. Poyatos[2,*]

[1] Instituto de Biología Molecular y Celular de Plantas, CSIC–UPV, 46022 Valencia, Spain

[2] Logic of Genomic Systems Laboratory, CNB–CSIC, 28049 Madrid, Spain

[*] jpoyatos@cnb.csic.es

Analysis of fluorescence data

We collected time-course data of fluorescence and absorbance and subtracted background values, which corresponded to M9 minimal medium, to correct the signals. The normalized fluorescence (for both YFP and CFP) was calculated as the ratio of fluorescence and absorbance. Similar values of normalized fluorescence were reported for MG1655 cells and for M9 minimal medium what indicated that the auto-fluorescence of cells was negligible in this case. The growth rate of cells was calculated as the slope of the linear regression between the log of corrected absorbance and time in exponential phase[1]. Time-dependent promoter activity, defined as the instantaneous production rate of normalized YFP fluorescence (magnitude per cell), was computed for each time point using the derivative of the normalized fluorescence. Promoter activity in steady state, defined as the stationary production rate of normalized YFP fluorescence (magnitude per cell) in

exponential phase, was calculated as the average over time (for $t > 2$ h) of normalized fluorescence times growth rate. Errors were obtained by calculating the standard deviation over replicates, in all time points, then squaring all these deviations to average them, and finally getting the root square. Data were analyzed with Matlab (MathWorks).

Bottom-up mathematical model

We developed a system of ordinary differential equations based on the known regulatory topology, and molecular details, of the MAR circuit. MarR, MarA and MarB constitute an operon whose expression is controlled by promoter P_{mar}[2,3]. MarR and MarB repress P_{mar}[4,5], with MarR action being reduced by salicylate, whereas MarA activates P_{mar}[6]. P_{mar} is further activated by Rob[2] (that represses its own promoter P_{rob}), while MarA represses in turn P_{rob}[7]. Finally, in our experimental system YFP models a downstream gene controlled by promoter P_{mar}. Although MarR, MarA and MarB are transcribed from the same promoter, the corresponding protein expressions may be different due to their individual translation rates. By analyzing the 5' untranslated regions of MarR, MarA and MarB with RBS calculator[8], considering the 30 nucleotides upstream and the 7 nucleotides downstream of the start codon, we obtained that translation rates of MarA and MarB are about 30-fold and 20-fold, respectively, higher than the translation rate of MarR. The difference agrees with previous experimental observations[9]. In addition, promoter P_{mar} is regulated by CRP:cAMP. Here, we do not consider the moderate activation of Rob by salicylate[3], because this protein is already highly expressed.

Therefore, we write

$$\begin{aligned}
&\frac{d}{dt}[MarA]=\beta\Pi_{mar}-(\mu+\delta)[MarA]\\
&\frac{d}{dt}[MarR]=\Pi_{mar}-\mu[MarR]\\
&\frac{d}{dt}[MarB]=\beta^{*}\Pi_{mar}-\mu[MarB]\\
&\frac{d}{dt}[Rob]=\Pi_{rob}-\mu[Rob]\\
&\frac{d}{dt}[YFP]=\Pi_{mar}-\mu[YFP],
\end{aligned} \qquad \text{(S1)}$$

where μ is the cell growth rate, δ the degradation rate of MarA ($\delta \gg \mu$, as MarA is quickly degraded by Lon protease[10]), β (and β^{*}) the fold increase of MarA (MarB) translation rate, and Π_{mar} and Π_{rob} the activity of promoters P_{mar} and P_{rob}, respectively.

Π_{mar} and Π_{rob} activity can be described in terms of Hill functions[11], in which MarA and Rob act as monomers whereas MarR functions as dimer[6]. The effect of MarA on P_{rob} is not observable in physiological conditions[3], so it is neglected to simplify the system of equations. Since MarB might not exert an observable repression on P_{mar} in presence of salicylate, it was also eliminated from the model. Π_{mar} reads then as

$$\Pi_{mar} = \Pi_0 \frac{1+f_a[MarA]/K_A+f_b Q([Rob],[cAMP])}{1+[MarA]/K_A+Q([Rob],[cAMP])} \frac{1}{1+([MarR_{free}]/K_R)^2}. \qquad \text{(S2)}$$

K_A, and K_R denote here the effective dissociation constants for transcription regulation, and f_a and f_b the activation fold changes. Π_0 is the basal protein synthesis rate, and Q a regulatory function that accounts for the activation of Rob and CRP:cAMP. Assuming competitive binding between the three transcriptional activators (MarA, Rob and CRP:cAMP), as their operators overlap, we can write

$$Q([Rob],[cAMP]) = ([Rob]/K_B)+([cAMP]/K_C), \quad \text{(S3)}$$

with K_B and K_C being the effective dissociation constants for transcription regulation. This model can explain that, in presence of Rob or cAMP, MarA does not increase significantly the occupancy of polymerase at the *marRAB* promoter, which agrees with previous experimental and theoretical data[12]. In addition, we have

$$[MarR_{free}] = \frac{1+\alpha([Sal]/\theta)^{\nu}}{1+([Sal]/\theta)^{\nu}}[MarR] \ , \quad \text{(S4)}$$

where θ is the effective dissociation constant between salicylate (Sal) and MarR, ν the Hill coefficient, and α a minimal fraction of free MarR.

Simplification of the mathematical model

The previous mathematical model can be simplified for a better analysis of the dynamics. We introduced the following normalized variables $x = [MarA]/K_R$, $y = [MarR]/K_R$, $y_0 = [MarR_{free}]/K_R$ (the concentration of MarR is equal to the one of YFP), $\rho = f_a = f_b$ (assuming for simplicity equal induction by the three transcriptional activators), $P_0 = \Pi_0/K_R$, and also $\kappa = K_R/K_A$ (K_A in the range 1000-1500 nM, and K_R around 5-10nM)[13,14]. We also denoted by z ($[Rob]/K_B$) and c ($[cAMP]/K_C$) the normalized concentrations of Rob and CRC:cAMP, respectively (and we considered $K_B = K_A$). Finally, time is given as number of cell cycles (in our experimental conditions, cell cycle of 99 min, for 5 mM salicylate). Thus, we obtained the model presented in the main text for the core (MarA, MarR) network

$$
\begin{aligned}
\frac{dx}{dt} &= \beta\pi_{mar} - \delta x \\
\frac{dy}{dt} &= \pi_{mar} - y \\
\pi_{mar} &= P_0 \frac{1+\rho(\kappa x + \kappa z + c)}{1+\kappa x + \kappa z + c} \cdot \frac{1}{1+{y_0}^2} .
\end{aligned} \qquad \text{(S5)}
$$

Here, MarA could be approximated to a quasi-steady state ($x \propto \pi_{mar}$, a function of time). And Eq. (S4) now reads

$$
\frac{y_0}{y} = \alpha_S = \frac{1+\alpha([Sal]/\theta)^\nu}{1+([Sal]/\theta)^\nu} \quad . \qquad \text{(S6)}
$$

In case of maximal induction of the system with salicylate, α modulates the regulatory role of MarR ($1 \geq \alpha_S \geq \alpha$, $\alpha = 1$ represents absence of salicylate effect).

Analytical solutions of the model: steady state

In this section, we considered that a very strong the repressor acted on the system, MarR (i.e., $y \gg 1$). We also assumed that the system was induced with high levels of salicylate, so that $y_0 = \alpha y$, and that the activation term was simply reduced to the fold change (ρ). This allowed us to simplify the model to just one equation, given by

$$
\frac{dy}{dt} \approx \frac{P_0 \rho}{\alpha^2 y^2} - y \quad . \qquad \text{(S7)}
$$

The steady state of this equation is given then by

$$
y_\infty \approx \left(\frac{P_0 \rho}{\alpha^2} \right)^{1/3} \quad . \qquad \text{(S8)}
$$

Analytical solutions of the model: dynamic range

Our model to describe the dynamics of a self-repressed, self-activated operon (*y*), implemented with two regulatory genes (repressor and activator), can be rewritten as

$$\frac{dy}{dt} = \frac{P_0}{1+(\alpha_S y)^2}\frac{1+\rho\kappa' y}{1+\kappa' y} - y, \tag{S9}$$

We used in this derivation Eq. (S6), and also assumed MarA to be a stable protein ($\delta = \mu$). This assumption leads to $x = \beta y$ and does not change the dynamics of our protein of interest, MarR (*y*). The simplified model can be solved in steady state for a strong repressor ($y \gg 1$) and high activation fold ($\rho \gg 1$) to obtain

$$y_\infty \approx \left(\frac{P_0 \rho}{\alpha^2}\right)^{1/3} \propto \alpha^{-2/3} \tag{S10}$$

For saturating levels of salicylate, we estimated with this model an output dynamic range of $R_{out} = \alpha^{-2/3}$ (i.e., the ratio between the highest and lowest values of y_∞ when varying salicylate), and an input dynamic range of $R_{\text{in}} = 9^{2/n_{\text{in}}}$ (i.e., the ratio between the salicylate values at which we have y_∞ and $y_\infty = max(y_\infty) - 0.1\Delta y_\infty$,where $\Delta y_\infty = max(y_\infty) - min(y_\infty)$; n_{in} is an effective Hill coefficient; see also Goldbeter & Koshland, 1981[15]). For $\alpha = 0.05$, it turned out $R_{\text{out}} = 7.37$ and $R_{\text{in}} = 28.32$ ($n_{\text{in}} = 1.31$).

Alternative models

For a constitutively expressed regulator (y), the dynamics of its regulated operon ($\bar{y}$) can be written as

$$\frac{d\bar{y}}{dt} = \frac{P_0\rho}{1+(\alpha_S y)^2} - \bar{y}, \tag{S11}$$

where y is constant. The steady state solution for $\bar{y}$ is straightforward to obtain.

Moreover, a model to describe the dynamics of a self-repressed gene (y), with equal production rate as before, can be written as

$$\frac{dy}{dt} = \frac{P_0\rho}{1+\left(\alpha_S y\right)^2} - y. \qquad \text{(S12)}$$

This model can be solved in steady state to obtain the same expression as before.

Finally, a model to describe the dynamics of a self-repressed gene (y) that becomes self-activator in presence of the inducer can be written as

$$\frac{dy}{dt} = P_0 \frac{1+\rho\omega\left(\left(1-\alpha_S\right)y\right)^n}{1+\omega\left(\left(1-\alpha_S\right)y\right)^n+\left(\alpha_S y\right)^2} - y, \qquad \text{(S13)}$$

where we have assumed competitive binding between the repressor and activator [an extra term $\Omega\omega\left(\left(1-\alpha_S\right)y\right)^n\left(\alpha_S y\right)^2$ in the denominator of (S13) includes the effect of independent binding, i.e., $\Omega = 0$ for competitive binding between oxidized and non-oxidized MarR, or $\Omega = 1$ for independent binding]. This model can be solved in steady state, also for a strong repressor ($y \gg 1$) and high activation fold ($\rho \gg 1$), as

$$y_\infty \approx P_0\rho\,\omega\,\theta_H\left(1-\alpha_S\right), \qquad \text{(S14)}$$

with θ_H representing the Heaviside function. Note that in the expressions above ω measures the asymmetry in the binding affinities of the two forms of MarR: $\omega = 1$ when binding is assumed similar (as in Fig. 6, main text), $\omega < 1$ for stronger binding of the oxidized form, and $\omega > 1$ when the non-oxidized form binding is stronger.). Moreover, the multimerization of the oxidized form could be considered to remain as a monomer ($n = 1$), dimer ($n = 2$), or tetramer ($n = 4$).

References

1. Rodrigo G., Bajic D., Elola I., and Poyatos J.F. Antagonistic autoregulation speeds up a homogeneous response in Escherichia coli. Sci Rep 6: 36196 (2016)
2. Alekshun M.N., Levy S.B. Regulation of chromosomally mediated multiple antibiotic resistance: the mar regulon*. Antimicrob Agents Chemother 41:* 2067-2075 (1997)
3. Chubiz L.M., Glekas G.D., Rao C.V. Transcriptional cross talk within the mar-sox-rob regulon in Escherichia coli is limited to the rob and marRAB operons. *J Bacteriol 194: 4867-4875* (2012)
4. Cohen S.P., Levy S.B., Foulds J., Rosner J.L. Salicylate induction of antibiotic resistance in Escherichia coli: activation of the mar operon and a mar-independent pathway. *J Bacteriol 175: 7856-7862* (1993)
5. Vinué L., McMurry L.M., Levy S.B. The 216-bp marB gene of the marRAB operon in Escherichia coli encodes a periplasmic protein which reduces the transcription rate of marA. *FEMS Microbiol Lett* 345: 49-55 (2013)
6. Martin R.G., Jair K.W., Wolf R.E. Jr., Rosner J.L. Autoactivation of the marRAB multiple antibiotic resistance operon by the MarA transcriptional activator in Escherichia coli. *J Bacteriol* 178: 2216-2223 (1996)
7. Schneiders T., Levy S.B. MarA-mediated transcriptional repression of the rob promoter. *J Biol Chem* 281: 10049-10055 (2006)
8. Salis H.M., Mirsky E.A., Voigt C.A. Automated design of synthetic ribosome binding sites to control protein expression. *Nat Biotechnol* 27: 946-950 (2009)
9. Martin R.G., Rosner J.L. Transcriptional and translational regulation of the marRAB multiple antibiotic resistance operon in Escherichia coli. *Mol Microbiol* 53: 183-191 (2004)
10. Griffith K.L., Shah I.M., Wolf R.E. Proteolytic degradation of Escherichia coli transcription activators SoxS and MarA as the mechanism for reversing the induction of the superoxide (SoxRS) and multiple antibiotic resistance (Mar) regulons. *Mol Microbiol* 51: 1801-1816 (2004)
11. Bintu L., Buchler N.E., Garcia H.G., Gerland U., Hwa T., Kondev J., Phillips R. Transcriptional regulation by the numbers: models. *Curr Opin Genet Dev* 15: 116-124. (2005)
12. Wall M.E., Markowitz D.A., Rosner J.L., Martin R.G. Model of transcriptional activation by MarA in Escherichia coli. *PLoS Comput Biol* 5: e1000614 (2009)
13. Martin R.G., Bartlett E.S., Rosner J.L., Wall M.E. Activation of the Escherichia coli marA/soxS/rob regulon in response to transcriptional activator concentration. *J Mol Biol* 380: 278-284 (2008)

14. Seoane A.S., Levy S.B. Characterization of MarR, the repressor of the multiple antibiotic resistance (mar) operon in Escherichia coli. *J Bacteriol* 177: 3414-3419 (1995)

15. Goldbeter A., Koshland D.E. Jr. An amplified sensitivity arising from covalent modification in biological systems. *Proc Natl Acad Sci USA* 78: 6840-6844 (1981)

SUPPORTING INFORMATION CAPTIONS

Figure S1: Architecture and input function of the *mar* operon *cis*-regulatory region. (a) Scheme of the *marRAB* promoter, showing the different operators according to RegulonDB (ref. 58 in main text). (b) Input function (promoter activity in steady state) as a function of the salicylate concentration (S) is represented for each system. Open circles correspond to experimental data (error bars are standard deviations of three replicates). Solid lines correspond to fittings to the model $[A_0 + A(S/K)^n] / [1 + (S/K)^n]$. Parameter A was fixed *a priori*. Representation in arbitrary units (AU).

Figure S2: Effect of MarB on the *mar* operon. (a) Comparison of the temporal responses of the WT (dashed lines) and Δ*marB* (solid lines) systems upon induction with two different salicylate concentrations. Normalized fluorescence (YFP) with time is represented (average of three replicates). (b) Characterization by qPCR of the intracellular RNA concentration of gene *marA* for the wild-type and Δ*marB* systems, with and without salicylate (5 mM). We report the ratio between the expression of gene *marA* and a constitutive housekeeping gene (16S rRNA). Error bars correspond to standard deviations of three replicates. Two-tailed, heteroscedastic, Student's *t*-tests were applied. Activation with salicylate for the WT and Δ*marB* systems, $P < 0.05$. Repression by MarB with and without salicylate, $P > 0.1$.

Figure S3: Temporal dynamics of the combinatorial regulation of the *mar* operon by salicylate and cAMP. (a) WT system. (b) Δ*rob* system.

Normalized fluorescence (YFP) with time is represented (average of three replicates).

Figure S4: Effect of MarR oxidation on the *mar* operon. (a) Transfer function of copper regulation relating the fraction of oxidized MarR ($MarR_{ox}$) with the relative amount of Cu^{2+}. Experimental values (small circles, data taken from previous work [38]) represent averages of replicates of the relative to the maximum oxidation. Here, we denote $U = [Cu^{2+}] / [MarR_{tot}]$ (intracellularly, $U \approx 5$ upon induction with salicylate). The line corresponds to $U^2 / (K_{ox}^2 + U^2)$, fitted with $K_{ox} = 1.35$. Hence, the remaining fraction of non-oxidized MarR upon fully induction is $\alpha \approx 5\%$. (b) Model simulation of how response time would change as a function of α (10 mM salicylate). The black point corresponds to the predicted value (with $\alpha = 0.05$). The gray point describes a limiting regime in which most MarR molecules are titrated (i.e., no repression). Inset shows a good correspondence between model predictions and experimental values (open circles) of response time. (c) Model simulation of the change in input (dashed) and output (solid) dynamic ranges of the system with α. Black points correspond to the experimental values reported for the WT system. Gray points correspond to the model predictions for the chosen parameterization. A vertical red line highlights the value of α used in this work.

Figure S5: Response of the *mar* operon with two different histories. (a, b) Single-cell response of initially uninduced, and initially fully induced,

populations [arbitrary units (AU)]. (c) Population temporal response. Normalized fluorescence (YFP) with time is represented (average of three replicates). Solid lines correspond to cells initially uninduced, whilst dashed lines to cells initially fully induced. Data for WT system.

Figure S6 Comparison of genetic implementations with more relaxed biochemical assumptions. (a-c) Model simulations of the response time (t_{50}), output dynamic range (R_{out}), and input dynamic range (R_{in}) as a function of the asymmetry in the binding affinities of MarR and $MarR_{ox}$ (ω,ω = 1 denotes similar binding, see Supplement), with competitive binding between MarR and $MarR_{ox}$. Different degrees of $MarR_{ox}$ multimerization were also studied (n = 1, 2, 4), (d-m) Model simulations of the dynamic response, with time (5 mM salicylate) and with salicylate, of alternative implementations of the *mar* core network (solid lines). Dashed lines correspond to the natural network. In both cases, we considered absence of Rob and CRP:cAMP. The width of the arrows represents asymmetry in the binding and the intensity of the gray color denotes non-oxidized/oxidized MarR.

Figure S7: Response time of a hypothetical dual regulator design of the *mar* core network. Curves denote model predictions of the response time as function of salicylate. Blue curve corresponds to the natural situation, in absence of Rob and CRP:cAMP. Gray curves denote a hypothetical system where the oxidized MarR acts as an activator. Solid gray curve for competitive binding between the repressor and activator, and dashed gray curve for independent binding.

Table S1: Nominal values of the parameters used in the model. Specific values use in the modeling of *Escherichia coli*'s *mar* response.

Table S2: *E. coli* operons with positive and negative autoregulation. Study of the operons from RegulonDB (ref. 58 in main text) that code for transcription factors acting as repressors and activators on their own promoter to reveal that the *mar* core network presents a unique genetic architecture.
