# Supplementary figures and images for: Deconstructing a multiple antibiotic resistance regulation through the quantification of its input function
Source: NPJ Syst Biol Appl. 2017 Oct 6;3:30. doi: 10.1038/s41540-017-0031-2 (PMC5630622; doi:10.1038/s41540-017-0031-2)

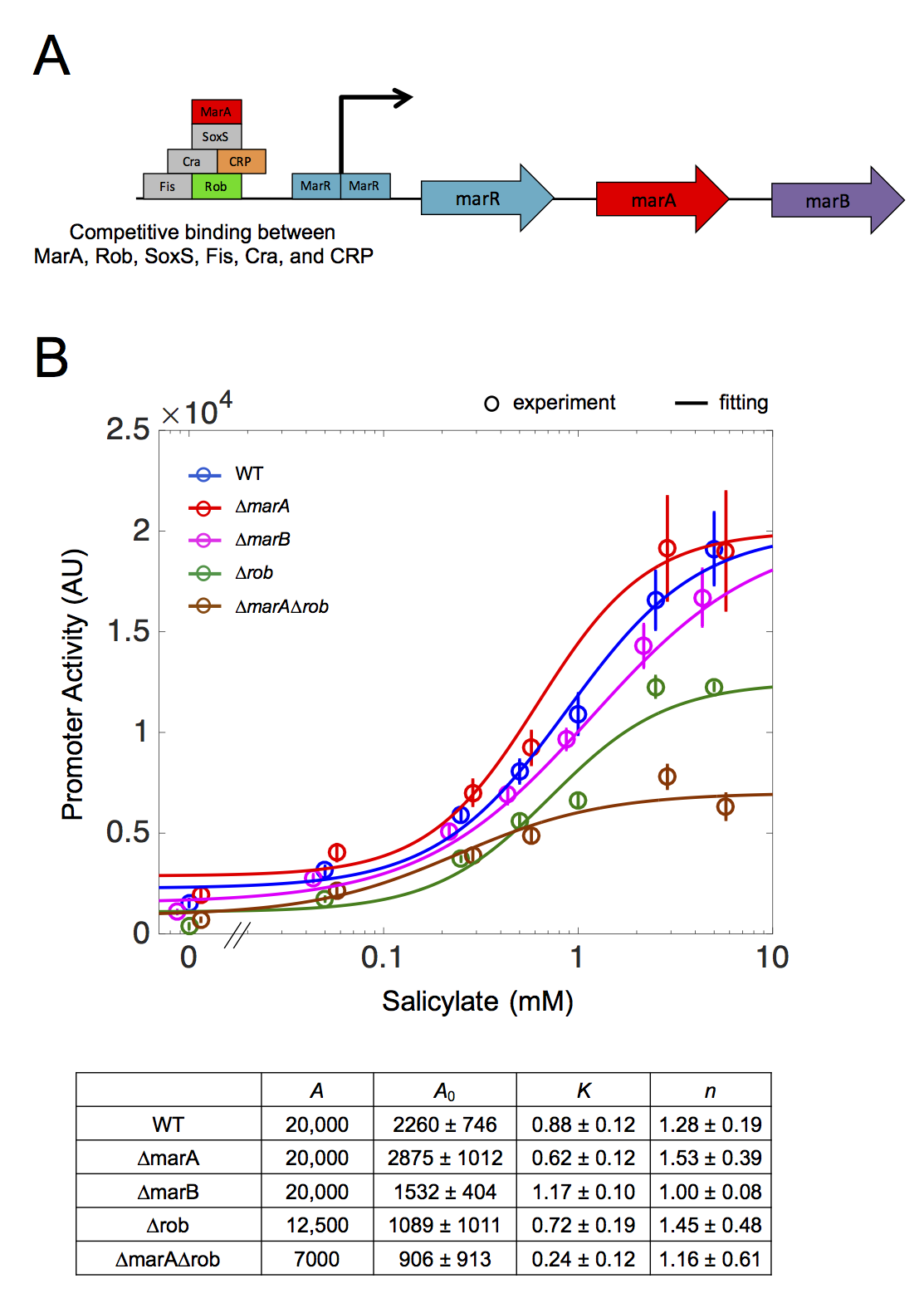

Supplement: Supplementary file 4 — Supplementary Figure S1 [file 41540_2017_31_MOESM4_ESM.tif]

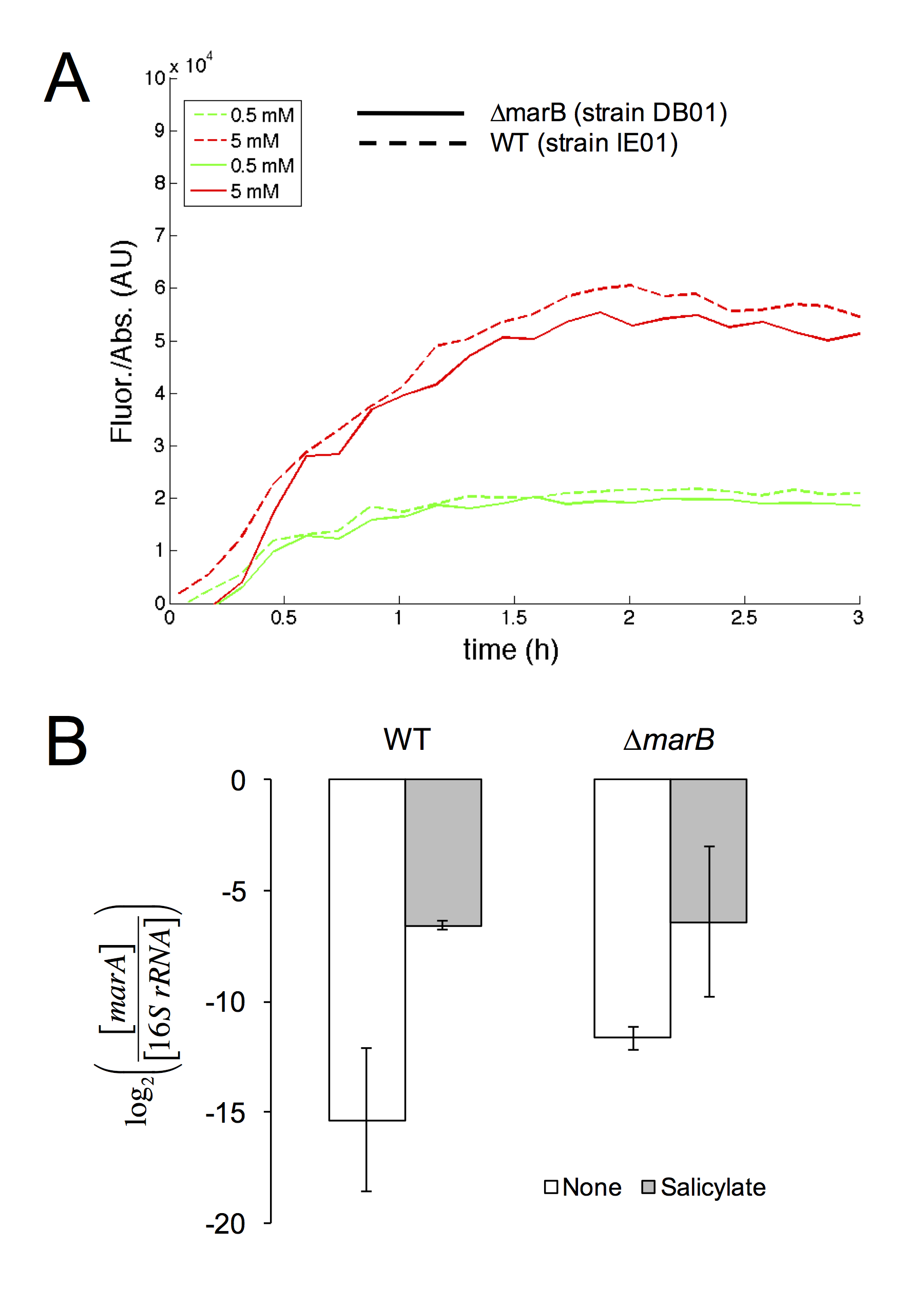

Supplement: Supplementary file 5 — Supplementary Figure S2 [file 41540_2017_31_MOESM5_ESM.tif]

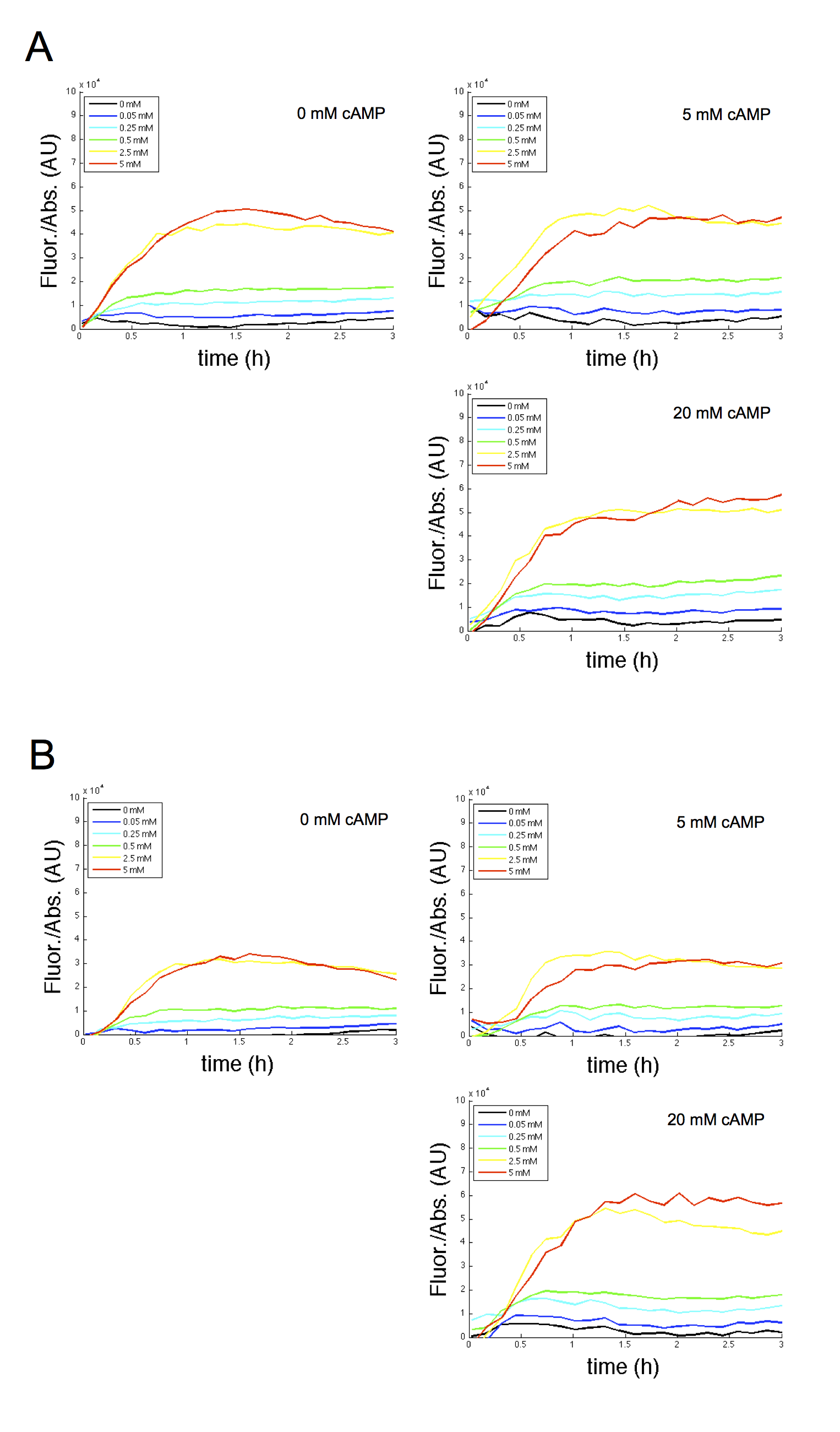

Supplement: Supplementary file 6 — Supplementary Figure S3 [file 41540_2017_31_MOESM6_ESM.tif]

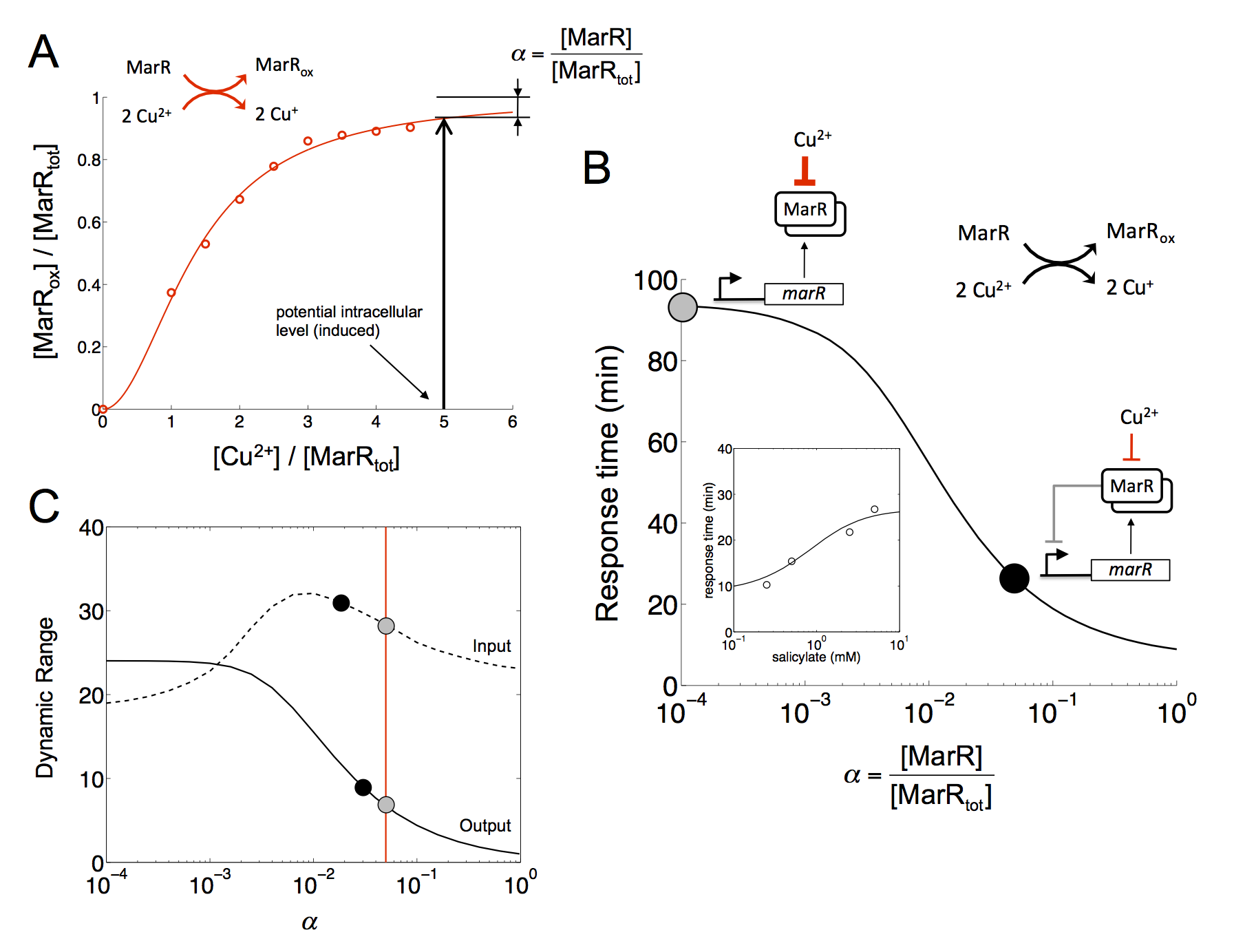

Supplement: Supplementary file 7 — Supplementary Figure S4 [file 41540_2017_31_MOESM7_ESM.tif]

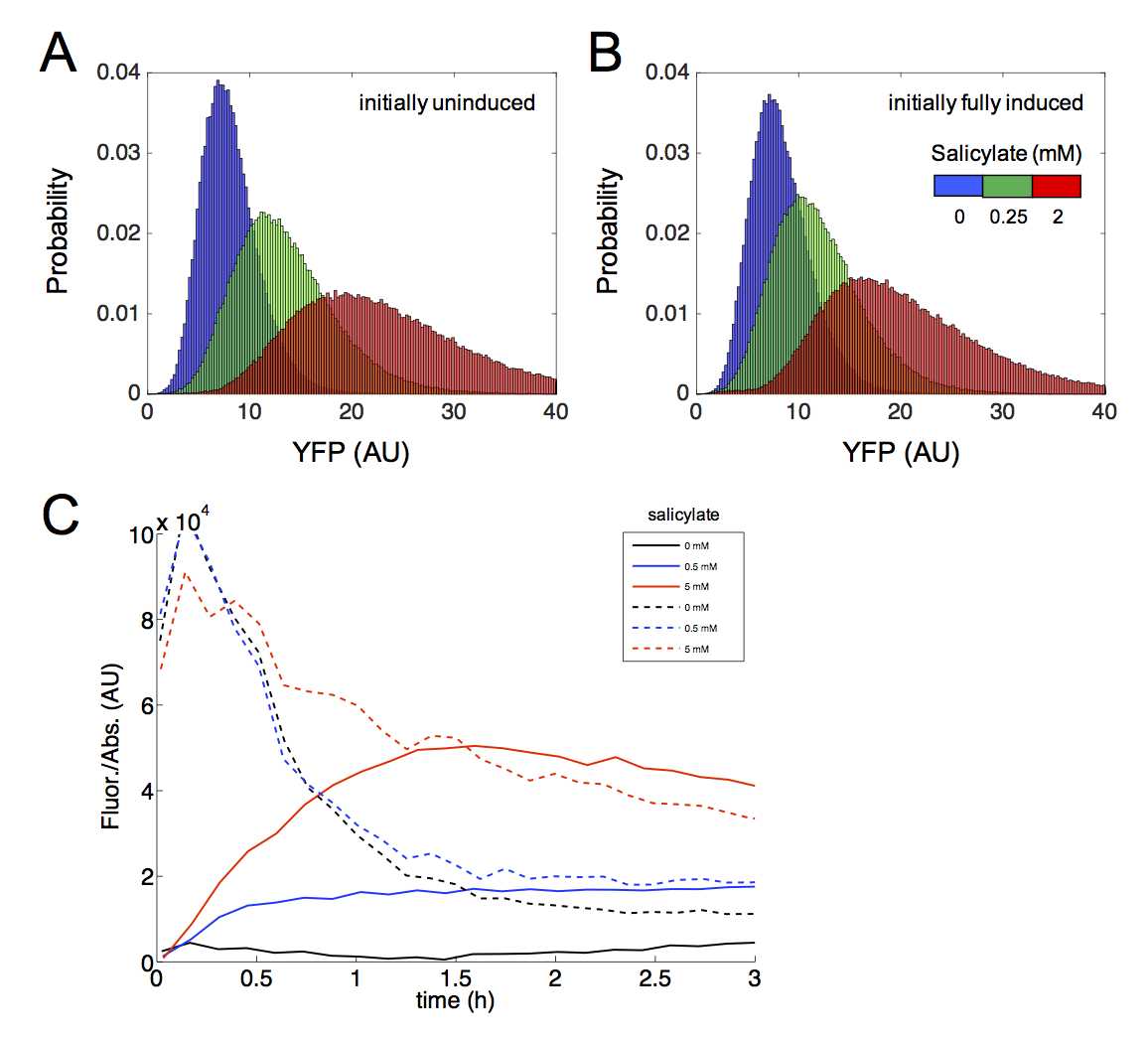

Supplement: Supplementary file 8 — Supplementary Figure S5 [file 41540_2017_31_MOESM8_ESM.tif]

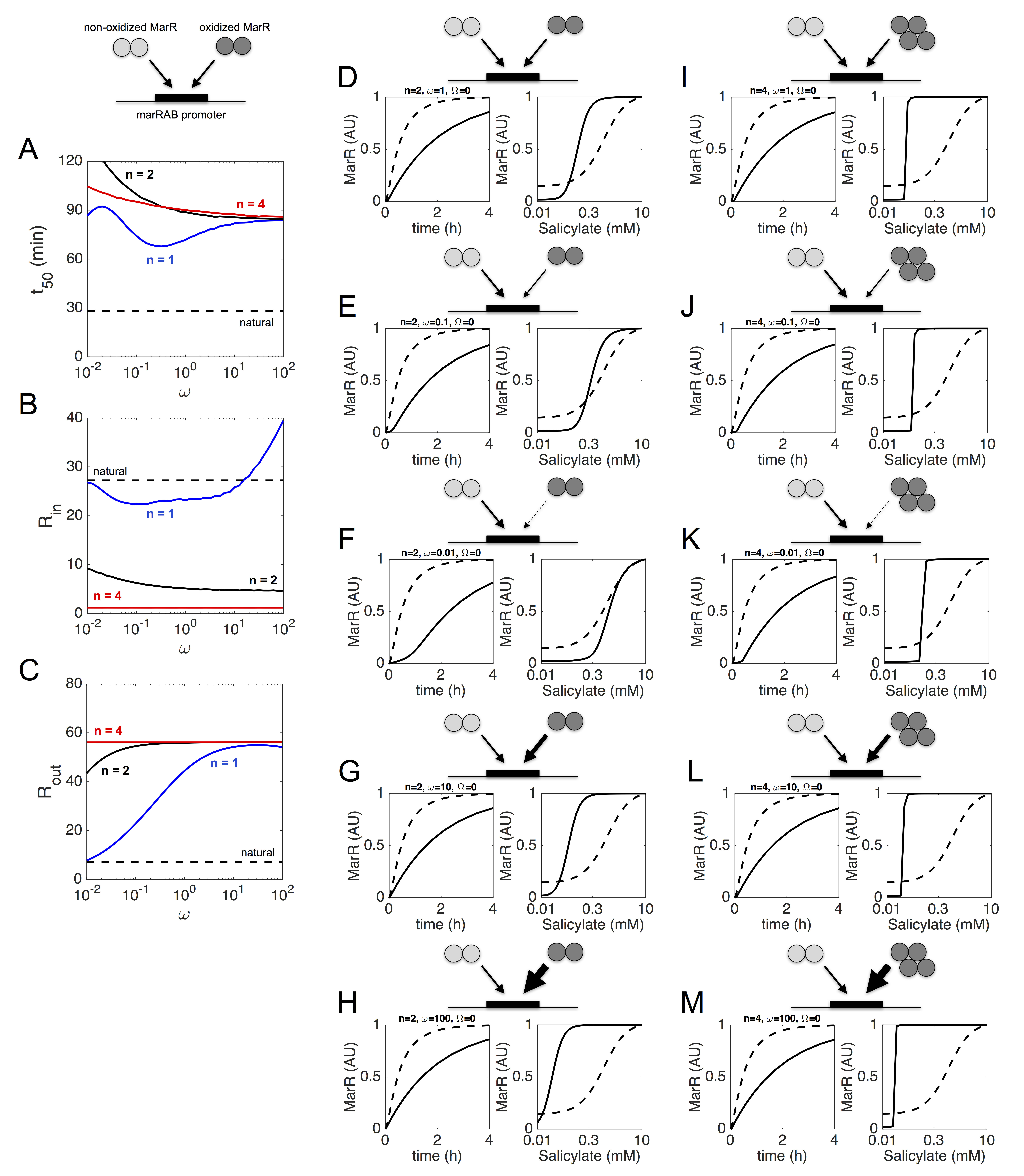

Supplement: Supplementary file 9 — Supplementary Figure S6 [file 41540_2017_31_MOESM9_ESM.tif]

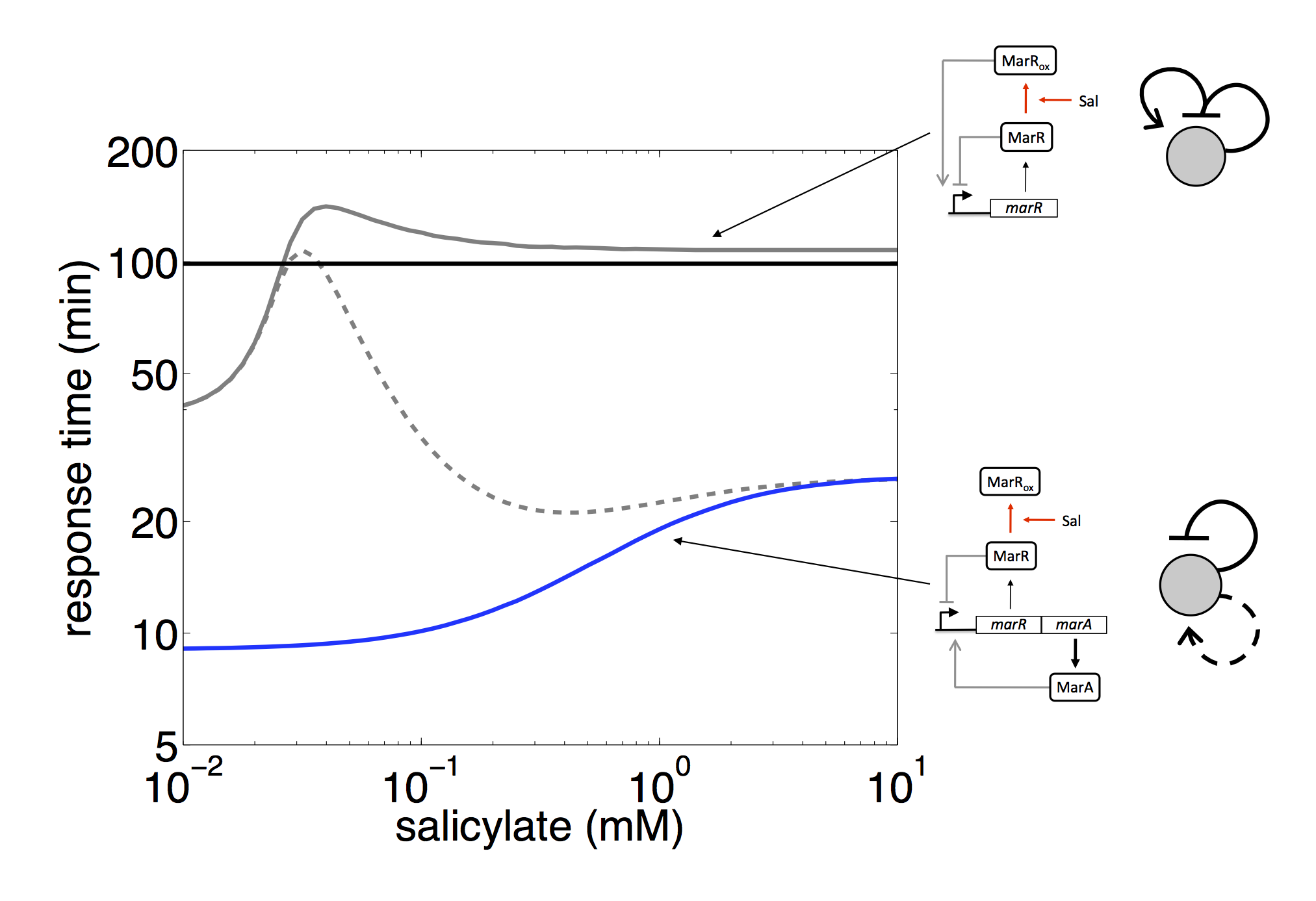

Supplement: Supplementary file 10 — Supplementary Figure S7 [file 41540_2017_31_MOESM10_ESM.tif]
